# PRMT1 is a novel molecular therapeutic target for clear cell renal cell carcinoma

**DOI:** 10.7150/thno.42345

**Published:** 2021-03-12

**Authors:** Jianfeng Wang, Chen Wang, Pan Xu, Xiao Li, Yongning Lu, Di Jin, Xiaomao Yin, Hao Jiang, Jing Huang, Huan Xiong, Fei Ye, Jia Jin, Yu Chen, Yiqian Xie, Zhifeng Chen, Hong Ding, Hao Zhang, Rongfeng Liu, Hualiang Jiang, Kaixian Chen, Zhiyi Yao, Cheng Luo, Yiran Huang, Yuanyuan Zhang, Jin Zhang

**Affiliations:** 1 Department of Urology, Renji Hospital, School of Medicine, Shanghai Jiaotong University, Shanghai 200127, China.; 2 Drug Discovery and Design Center, CAS Key Laboratory of Receptor Research, State Key Laboratory of Drug Research, Shanghai Institute of Materia Medica, Chinese Academy of Sciences, 555 Zuchongzhi Road, Shanghai 201203, China.; 3 University of Chinese Academy of Sciences, 19 Yuquan Road, Beijing 100049, China.; 4 Zhongshan hospital Fudan University, 180 Fenglin road, Shanghai 200032, China.; 5 Department of Chemistry, College of Sciences, Shanghai University, 99 Shangda Road, Shanghai 200444, China.; 6 Department of Medicinal Chemistry, Shanghai Institute of Materia Medica, Chinese Academy of Sciences, 555 Zuchongzhi Road, Shanghai 201203, China.; 7 College of Life Sciences, Zhejiang Sci-Tech University, Hangzhou 310023, China.; 8 PerkinElmer Management (Shanghai) Co. Ltd, 1670 Zhangheng Road, Zhangjiang High-Tech Park, Shanghai 201203, China.; 9 In vitro Biology, Shanghai ChemPartner Life Science Co., Ltd., #5 Building, 998 Halei Road, Shanghai 201203, China.; 10 College of Chemical and Environmental Engineering, Shanghai Institute of Technology, Shanghai 210032, China.

**Keywords:** clear cell renal cell carcinoma, protein arginine methyltransferase 1, therapeutic, lipocalin2 (LCN2), sunitinib

## Abstract

**Background and Objective:** Epigenetic alterations are common events in clear cell renal cell carcinoma (ccRCC), and protein arginine methyltransferase 1 (PRMT1) is an important epigenetic regulator in cancers. However, its role in ccRCC remains unclear.

**Methods:** We investigated PRMT1 expression level and its correlations to clinicopathological factors and prognosis in ccRCC patients based on ccRCC tissue microarrays (TMAs). Genetic knockdown and pharmacological inhibition using a novel PRMT1 inhibitor DCPT1061 were performed to investigate the functional role of PRMT1 in ccRCC proliferation. Besides, we confirmed the antitumor effect of PRMT1 inhibitor DCPT1061 in ccRCC cell-derived tumor xenograft (CDX) models as well as patient-derived tumor xenograft (PDX) models.

**Results:** We found PRMT1 expression was remarkably upregulated in tumor tissues and associated with poor pathologic characters and outcomes of ccRCC patients. Furthermore, genetic knockdown and pharmacological inhibition of PRMT1 by a novel potent inhibitor DCPT1061 dramatically induced G1 cell cycle arrest and suppressed ccRCC cell growth. Mechanistically, RNA sequencing and further validation identified Lipocalin2 (LCN2), a secreted glycoprotein implicated in tumorigenesis, as a crucial regulator of ccRCC growth and functional downstream effector of PRMT1. Epigenetic silencing of LCN2 autocrine secretion by PRMT1 deficiency decreased downstream p-AKT, leading to reduced p-RB and cell growth arrest through the neutrophil gelatinase associated lipocalin receptor (NGALR). Moreover, PRMT1 inhibition by DCPT1061 not only inhibited tumor growth but also sensitized ccRCC to sunitinib treatment *in vivo* by attenuating sunitinib-induced upregulation of LCN2-AKT-RB signaling.

**Conclusion:** Taken together, our study revealed a PRMT1-dependent epigenetic mechanism in the control of ccRCC tumor growth and drug resistance, indicating PRMT1 may serve as a promising target for therapeutic intervention in ccRCC patients.

## Introduction

Renal cell carcinoma (RCC), which arises from renal tubular epithelial cells, is the most common type of kidney cancer in adults with nearly 73,820 new cases and 14,770 deaths occurred in the USA 1. Among all the histopathological subtypes of RCC, clear cell renal cell carcinoma (ccRCC) is the predominant subtype that accounts for more than 80% of RCC 2. Although surgical excision of tumors could cure most early-stage ccRCC, patients with recurrence or metastasis still face limited treatment options 3. Sunitinib, as well as several other tyrosine kinase inhibitors (TKIs), has been approved as the first-line therapy for patients with advanced ccRCC. However, the benefit is rather limited due to drug resistance and unique toxicity profiles. Checkpoint inhibitors, such as monoclonal antibodies that target programmed cell death protein 1 (PD-1) or cytotoxic T-lymphocyte-associated antigen 4 (CTLA-4), have emerged as novel therapeutic options with durable but limited responses 4. Overall, none of these therapeutic methods show an excellent antitumor effect for advanced ccRCC patients. More recently, combination therapy has become a hot spot in sensitizing TKIs in clinical trials, which may provide new opportunities to identify effective therapeutic strategies for ccRCC 5. Therefore, the development of novel treatment as a monotherapy or in combination with TKIs is in urgent need for ccRCC patients.

Epigenetic alterations are essentially involved in tumor progression and are known to be pharmacologically reversible, which makes them an attractive therapy for anticancer drug development 6. Accumulating evidence shows that changes in DNA methylation, microRNA and post-translational histone modifications occurred in several crucial signaling pathways in ccRCC, including VHL-HIF, WNT-β-catenin and EMT pathways 7, 8. The involvement of DNA methylation and microRNA in ccRCC has been well characterized, while alterations of histone modification in ccRCC remain to be investigated 7, 9. Among these epigenetic alterations, protein arginine methylation is reported to play a critical role in normal and pathological development 10, 11. Protein arginine methyltransferase 1 (PRMT1), the predominant type I PRMT, catalyzes ω-NG-monomethylation (MMA) and ω-NG, NG-asymmetric dimethylation (ADMA). PRMT1 has been reported to regulate various biological processes including RNA metabolism, genome stability maintaining, signal transduction, as well as gene transcription 12, 13. Misregulation of PRMT1 has been implicated in the progression of various diseases, especially cancer 14. For example, in hematological and solid tumors like lung and colon cancer, PRMT1 promotes tumor growth 15, 16. While in non-MYCN amplified neuroblastoma, PRMT1 plays a tumor-suppressive role in cancer progression 17. Importantly, Filipovic J and her colleagues reported PRMT1 may act as a tumor suppressor gene by analyzing the expression profile of PRMT1 in ccRCC tumor samples 18. However, multiple functional experiments with ccRCC tissues and cells are needed to investigate the exact role of PRMT1 in ccRCC. Besides, pharmacological intervention using PRMT1 inhibitor will be helpful to assess whether PRMT1 can serve as a therapeutic target for cancer treatment.

In this study, we reported the overexpression of PRMT1 in ccRCC tumors and its correlations to clinicopathological factors and prognosis in ccRCC patients. The essential role of PRMT1 in the proliferation and cell cycle of ccRCC cells was unraveled using a combinatorial approach of genetic knockdown and pharmacological inhibition by a novel PRMT1 inhibitor DCPT1061. We further revealed that Lipocalin2 (LCN2), a crucial regulator for ccRCC growth, functioned as a downstream effector of PRMT1. It has been reported that LCN2 promoted human pulmonary artery smooth muscle cell proliferation and triple-negative breast cancer cell invasion by activating the downstream AKT signaling 19, 20. Since the AKT signaling pathway is a critical signaling pathway in ccRCC which regulates G1 cell cycle progression by regulating RB phosphorylation 21, 22, we examined the effect of PRMT1 deficiency on the LCN2-AKT-RB signaling pathway. LCN2, also known as neutrophil gelatinase-associated lipocalin (NGAL), is an autocrine secreted glycoprotein involved in multiple processes, such as immunity, renal development, and apoptosis 23, 24. Furthermore, LCN2 has been reported to participate in multiple pathological processes, including inflammation, kidney injury, and especially tumorigenesis 25-27. PRMT1 knockdown or inhibition with DCPT1061 reduced the transcription of LCN2 by decreasing PRMT1-mediated H3R4me2a modification at the promoter region of the *LCN2* gene. We also demonstrated that DCPT1061 inhibited tumor growth and sensitized ccRCC to sunitinib treatment in ccRCC cell-derived tumor xenograft (CDX) models and patient-derived tumor xenograft (PDX) model. This study increased our understanding of the role of PRMT1 in ccRCC prognosis and progression, and suggested that PRMT1 inhibition may provide a promising targeted strategy for ccRCC treatment.

## Methods

### Patients and tissue samples

358 human ccRCC and corresponding adjacent non-tumorous tissue samples for tissue microarrays (TMAs) were collected from patients who underwent nephrectomy in Renji Hospital of Shanghai Jiatong University from January 2001 to December 2008. Besides, 39 paired ccRCC tumor specimens were conserved in liquid nitrogen for RT-qPCR and Western blot experiments, and one ccRCC tumor tissue was chosen for the xenograft experiment (PDX#1002523691). The pathologic diagnosis of all patients was determined by two experienced pathologists and all samples were confirmed as ccRCC. Comprehensive clinicopathologic information of patients, including gender, age, TNM stage, pathological grade, tumor size, and survival outcomes, were collected during the follow-up after surgery. The clinical stages were classified according to the 8^th^ TNM classification system, and the pathological grades were evaluated according to the WHO/ISUP 2016 grading system. Overall survival (OS) was calculated from the date of surgery to the latest follow-up or the day of death for any reason while recurrence-free survival (RFS) was calculated from the time of nephrectomy to the time of recurrence. Follow-ups were finished on Apr. 30, 2016, and the median overall survival was 106 months (ranging from 1 to 196 months). RNA sequencing data (RNAseqv2) of ccRCC patients from the Cancer Genome Atlas (TCGA, https://cancergenome.nih.gov/) was also used to assess the correlation of PRMT1 expression with patients' survival. We defined the PRMT1 expression value of 10.7 as the cutoff value for low and high expression with X-tile software according to the method described previously 28. This study was approved by the Ethics and Research Committees of Renji Hospital, Shanghai Jiao Tong University School of Medicine. Tissue samples were obtained with written consent from all the patients.

### RNA extraction and quantitative RT-PCR

TRIZOL reagent (Invitrogen) was used to isolate the total RNA of ccRCC tumor samples, and RNA was converted into cDNA with a special cDNA synthesis kit (Promega) according to the manufacturer's protocol. Human gene expression was measured using RT-qPCR on the ABI ViiA™ 7 System (America). Expression of target genes was normalized with the expression of β-ACTIN. The primer sequences were listed in **Supplementary Table S1**.

### Western blot analysis

Protein lysates were obtained from frozen tissue samples and cultured cells using RIPA buffer supplemented with protease and phosphatase inhibitors. After quantified, equal amounts of proteins were separated by SDS-PAGE and transferred onto a nitrocellulose membrane (Millipore, Temecula, CA, USA). Membranes were blocked with 3% BSA in PBST and incubated overnight at 4 °C with primary antibodies. After incubated with horseradish peroxidase-conjugated secondary antibodies (Abcam; Cambridge, UK), target protein bands were visualized using the enhanced chemiluminescence method in a ChemiScope3400 imaging system. Primary antibodies used were listed in **Supplementary Table S2**.

### Immunohistochemical analysis

TMAs were constructed according to the conventional protocol. Briefly, after tissue samples (tumor tissues and paired adjacent normal tissues) were collected and blocked, hematoxylin-eosin (HE) staining was performed by two experienced pathologists to define and mark the region of representative tissues for coring, and regions of artifact or necrosis were excluded. Then, the marked region of tissues was punched out from the donor paraffin block and regularly transferred into the blank recipient paraffin block according to the array design. After embedded, paraffin sections with a thickness of 4 μm were cut and transferred to anti-slip slides. Before immunohistochemical staining, the TMAs were first stained HE to confirm the right tissue cores were selected and the tissue shedding rate ≤ 5% was considered as a qualified TMAs. A total of 234 cores of paired adjacent normal tissues were included in the TMAs. Immunohistochemical staining was performed according to the conventional streptavidin-peroxidase method of immunohistochemistry (Zymed, San Francisco, USA). Primary antibodies were applied for immunohistochemistry (IHC) staining. Nikon Eclipse Ti Microscope (Nikon Corporation, Tokyo, Japan) and Leica DM6000 B (Leica Microsystems, Wetzlar, Germany) were used to record the results. Two uropathologists who did not know the information of the patients independently analyzed the immunohistochemistry results. Cells in five areas with the greatest amount of PRMT1 positive stains were selected to count and estimated at high (× 200) magnification. Then, the percentage of positive cells was scored as: 1 (0 - 25%), 2 (26 - 50%), 3 (51 - 75%), or 4 (> 75%). The intensity of positive staining was scored into the following four categories: 0 (negative), 1 (weak), 2 (moderate), or 3 (strong). Comprehensive score = staining percentage × intensity. Finally, we defined the PRMT1 expression level as follows: low expression: comprehensive score < 6; high expression: comprehensive score ≥ 6.

### Cell lines and cell culture

Cell lines 786-O, Caki-1, A498, and ACHN were purchased from the ATCC (American type culture collection). 786-O and Caki-1 cells were cultured in 1640 media with 10% heat-inactivated fetal bovine serum (FBS, Gibco, Australia). A498 and ACHN cells were cultured in MEM media with 10% FBS. Cells were maintained at 37 °C with 5% CO_2_, and cells with fewer than 50 passages were used for experiments.

### Lentiviral vectors construction and transfection

We produced lentiviral particles as previously described 29. Briefly, PRMT1 shRNA sequences and a control shRNA were integrated into the pSicoR backbone, and the shRNA-pSicoR plasmid was transfected in 293T cells. pSicoR was a gift from Tyler Jacks (Addgene plasmid #11579) 30. Supernatant with lentiviral particles was collected and filtered at 48 and 72 h after transfection. Human full-length LCN2 cDNA was inserted into the lentivirus vector pLent-EF1a-FH-CMV-GFP-P2A-Puro to construct LCN2 lentiviral vectors. Cells of ccRCC were instantly infected with lentiviral particles in the presence of 10 μg/mL Polybrene. At a particular time, ccRCC cells were collected for subsequent experiments. Primer sequences for PRMT1 knockdown were as previously described 29.

### Cell survival assay

After treatment, ccRCC cells were plated in 96-well plates and the cells were incubated overnight at 37 °C. Cell survival was determined by SRB assay according to standard protocol. Cell viability was estimated at an absorbance of 580 nm at different time points. All experiments were repeated three times.

### Cell cycle analysis

For cell cycle analysis, cell flow cytometry was used to examine ccRCC cells. Briefly, cells were collected and fixed in 70% ethanol at 4 °C overnight followed by staining with propidium iodide (PI), and were analyzed using FACS flow cytometer (Becton-Dickinson, Mountain View, CA). For each sample, 20,000 cells were analyzed with CellQuest software (Becton-Dickinson). Cell cycle distribution was analyzed and cells in G1, S, or G2/M-phase were counted both by ModFit software.

### Colony formation assay

Colony formation assay was performed to determine the colony-formation ability of tumor cells. Briefly, after treatment, cells were seeded into six-well plates with a low density. 10 days later, the colonies were washed, fixed, stained, photographed, and counted.

### siRNA interference for genes silence

Before the experiment, ccRCC cells were seeded into six-well plates with an appropriate density. Small interfering RNAs (siRNA) with lipofectamine RNAiMAX reagent (Invitrogen, Carlsbad, CA, USA) were transfected into ccRCC cells. 48 h later, western blot was used to detect the efficiency of gene knockdown. Sequence-specific siRNAs were listed in **Supplementary Table S3**.

### Xenograft model and treatments

Around 5 × 10^6^ ccRCC cells (Caki-1, A498) were injected subcutaneously into the flank region of six-week-old female nude mice (BALB/c-nu/nu). The PDX was established in six-week-old female SCID mice using the PDX#1002523691 tissue. Tumor volume was measured with a caliper, and the estimated tumor volume = length × width^2^ /2. When tumors reached approximately 50 mm^3^, four groups (*n* = 6) were divided randomly. Treatment of mice was as follows: vehicle control, DCPT1061 (30 mg/kg/day), sunitinib (25 mg/kg/day, purchased from MedChemExpress), and a combination of DCPT1061 (30 mg/kg/day) and sunitinib (25 mg/kg/day). Body weights and tumor volumes were measured every second day. After treatment, mice were sacrificed and the tumors were harvested, weighed, fixed with 4% formaldehyde. This study was approved by the Institute Animal Care and Use Committee at Shanghai Institute of Material Medica (2017-08-LC-02).

### RNA-sequencing analysis

The cultured cells were treated with DCPT1061 or shPRMT1 lentivirus respectively *in vitro* in 10cm dish plates. 48 h later, cells were collected, and total RNA was isolated. The well-constructed cDNA libraries were then sequenced on the Illumina HiSeq2000 using paired-end methods. After careful quality control of raw reads including trimming adapter sequences and filtering raw reads with low quality, the clean reads were then analyzed through a routine RNA sequencing analysis pipeline. The sequencing reads were first aligned to human hg19 genome by STAR 2.5 (Spliced Transcripts Alignment to a Reference) and then featureCounts software was used to quantify gene expression 31. Based on these raw counts, differential gene expression analysis was conducted using R/Bioconductor package DESeq2 32. To define genes differentially expressed in each sample, both fold change of 2 and an adjusted *P*-value of 0.05 were set as the cut-off value in each case.

### Chromatin immunoprecipitation analyses

We purchased the ChIP Kit from Cell Signal Technology (Cat No. #9005) and experiments were performed following the manufacturer's protocol. Antibody against H4R3me2a was bought from Active Motif (Cat No. 39705), and the primers for the LCN2 promoter were listed in **Supplementary Table S4**.

### Statistics

Statistical analyses were performed using SPSS 22 software, R software, or Graphpad Prism 6.0. The chi-square test and Fisher's exact test were performed to investigate the correlations between PRMT1 expression level and clinicopathologic characteristics, as well as OS and RFS. Survival curves were analyzed by the Kaplan-Meier method and compared with the log-rank test. Significant variables in univariate analysis were further analyzed by multivariate cox analysis to test for independent prognosis of ccRCC. R software with the “rms” package was used to analyze the nomograms and calibration plots. Significant parameters in multivariate analysis were integrated to construct nomograms. Data were presented as mean ± SD. Student's t-test was performed to calculate the statistical significance of differences between groups. Combination index (CI) was calculated using the CompuSyn software (Combo Syn, Inc., Paramus, NJ). CI > 1.0 antagonism, CI = 1.0 additive effect, CI < 1.0 indicates synergism. All statistical tests were two-sided. * *P* < 0.05, ** *P* < 0.01, *** *P* < 0.001.

## Results

### Increased PRMT1 expression correlates with the progression and prognosis of ccRCC patients

**Figure 1:**
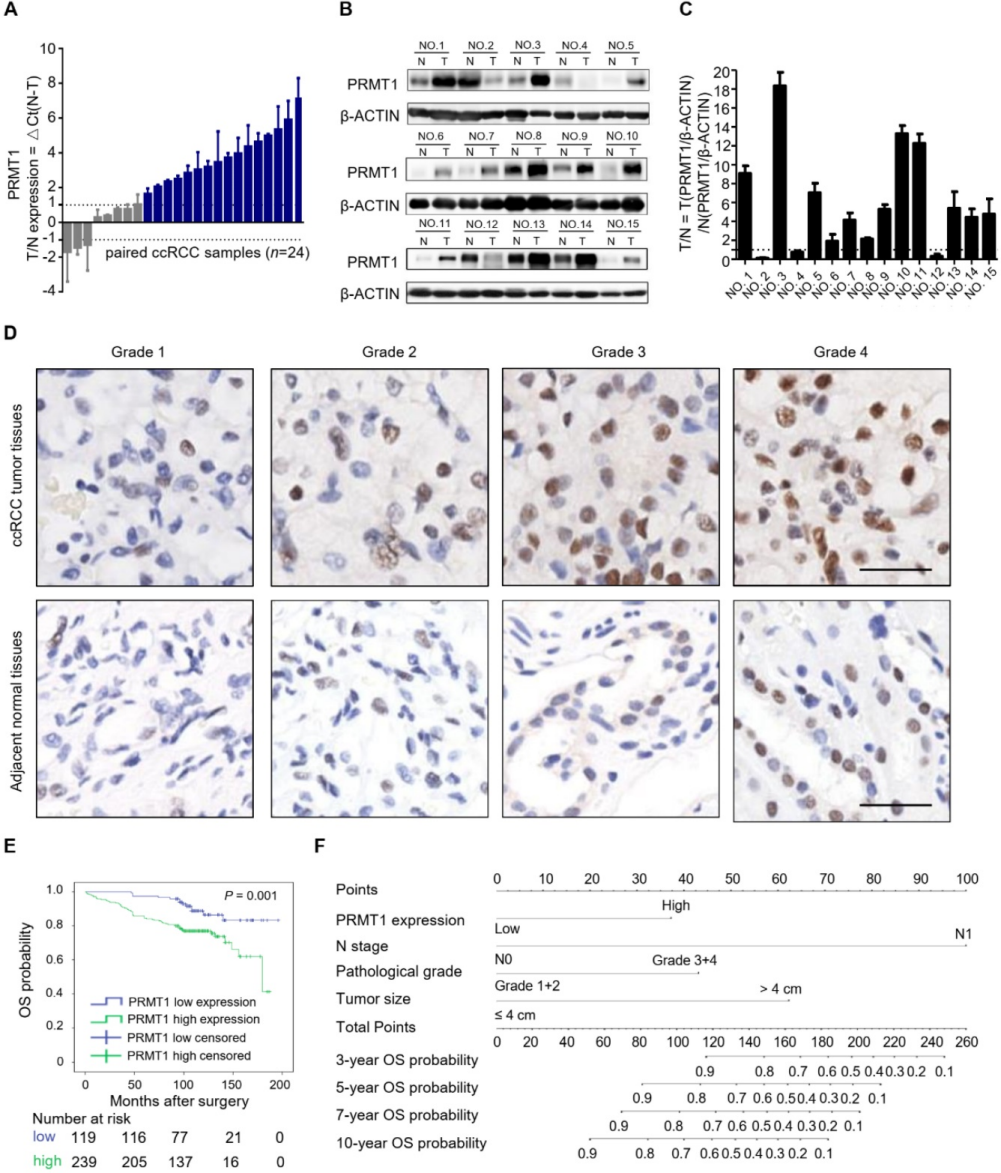
**High expression of PRMT1 correlates with tumor progression and shortened survival in ccRCC patients. (A)** Quantitative RT-PCR experiments for PRMT1 mRNA level in 24 pairs of human clinical ccRCC tumor tissues and adjacent normal tissues. **(B-C)** Western blot analysis for PRMT1 protein level from 15 pairs ccRCC tissue samples, and β-ACTIN was used as control. **(D)** Representative IHC micrographs of PRMT1 expression in different pathological grades of ccRCC patients from TMAs. Scale bar: 100 µm. **(E)** Kaplan-Meier curve of comparing OS in different levels of PRMT1 expression groups (*n*=119 in the low-expression group; *n*=239 in the high-expression group). **(F)** Nomogram for the prognosis of OS in ccRCC patients from TMAs.

In our search for potential epigenetic regulators of ccRCC progression, we noticed that PRMT1 and PRMT7 expression levels were significantly correlated with the progression and prognosis of ccRCC patients from TCGA (**Supplementary Figure S1**). However, the role of PRMT7 in ccRCC has been well investigated, while the functional role of PRMT1 in ccRCC progression remains poorly investigated 33. Thus, we mainly focused on studying PRMT1 in ccRCC. Firstly, we examined the expression of PRMT1 in ccRCC tumor tissues. Quantitative reverse transcription polymerase chain reaction (RT-qPCR) experiments were performed with 24 pairs of human ccRCC tissues and their matched normal tissues. 16 of 24 (66.7%) tumor samples showed increased mRNA levels of PRMT1, compared to the adjacent normal tissues (**Figure 1A**). The enhanced expression of PRMT1 was further confirmed at the protein level by Western blot analysis from 15 pairs of ccRCC tissue samples (**Figure 1B-C**). These data suggested that elevated expression of PRMT1 is a common event in ccRCC, which may play an important role in ccRCC oncogenesis.

To further investigate the potential clinical relevance of PRMT1 expression in ccRCC patients, immunohistochemistry (IHC) analysis was performed in ccRCC TMAs. Results consistently revealed that PRMT1 was significantly upregulated in 66.8% (239/358) of tumors, while the adjacent normal tissues exhibit upregulation of PRMT1 in only 38.5% of samples (**Supplementary Table S5**). As shown in **Table 1**, PRMT1 expression level was positively related to lymph nodes metastasis (*P* = 0.032) and pathological grade (*P* = 0.027). PRMT1 was significantly increased in grade III and IV compared with grades I and II (**Figure 1D**), suggesting that PRMT1 overexpression correlated with high pathologic grade of ccRCC. Furthermore, Kaplan-Meier method with log-rank test revealed that high expression of PRMT1 was significantly correlated with shortened OS and RFS of ccRCC patients (**Figure 1E; Supplementary Figure S2A**). Consistently, analysis of data from TCGA also showed that PRMT1 expression level significantly correlates with tumor progression of ccRCC, which conforming our findings (**Supplementary Figure S2B-C**). These findings together suggest that PRMT1 expression is significantly associated with ccRCC progression and may serve as a prognostic biomarker for ccRCC patients.

### PRMT1 expression is an independent prognostic factor for ccRCC patients

To investigate the clinical significance of PRMT1 for postoperative outcomes among patients with ccRCC, we further performed univariate and multivariate statistical analysis in our ccRCC cohort. In the multivariate cox analysis, patients with higher PRMT1 expression showed significantly shortened OS (HR, 2.493; *P* = 0.004; **Table 2**) and RFS (HR, 2.735; *P* = 0.002; **Table 2**) compared with their counterparts. It was also confirmed that lymph nodes metastasis (N stage, *P* = 0.011), pathological grade (*P* = 0.001) and tumor size (*P* < 0.001) were independent prognostic factors for OS, while lymph nodes metastasis (N stage, *P* = 0.013), pathological grade (*P* = 0.004) and tumor size (*P* < 0.001) were independent prognostic factors for RFS in ccRCC (**Table 2**).

Moreover, we built two prognostic nomograms for OS and RFS in ccRCC patients whose *P*-value < 0.05 in multivariate cox regression analysis via integrating all the independent prognostic factors (**Figure 1F; Supplementary Figure S2D**). The survival probabilities of ccRCC patients at different time points after surgery could be predicted by total points which were calculated by adding up each point of each parameter 34. The calibration plots at 3, 5, 7, and 10 years after surgery showed good consistency between actual observation and the prediction by prognostic nomograms (**Supplementary Figure S3**). Moreover, to further evaluate the predictive ability of all of these independent prognostic factors, Harrell's concordance index (C-index) value and Akaike information criterion (AIC) value were calculated 35. Results indicated that PRMT1 exhibited better prognoses when combined with other prognostic factors than without PRMT1 integrated models in predicting OS and RFS of ccRCC (**Table 3**). Our data indicated PRMT1 expression level is an independent prognostic factor, and when integrated with several conventional features it could predict the prognosis possibility of ccRCC patients.

### Knockdown of PRMT1 impairs cellular proliferation and cell cycle progression of ccRCC cells

**Figure 2:**
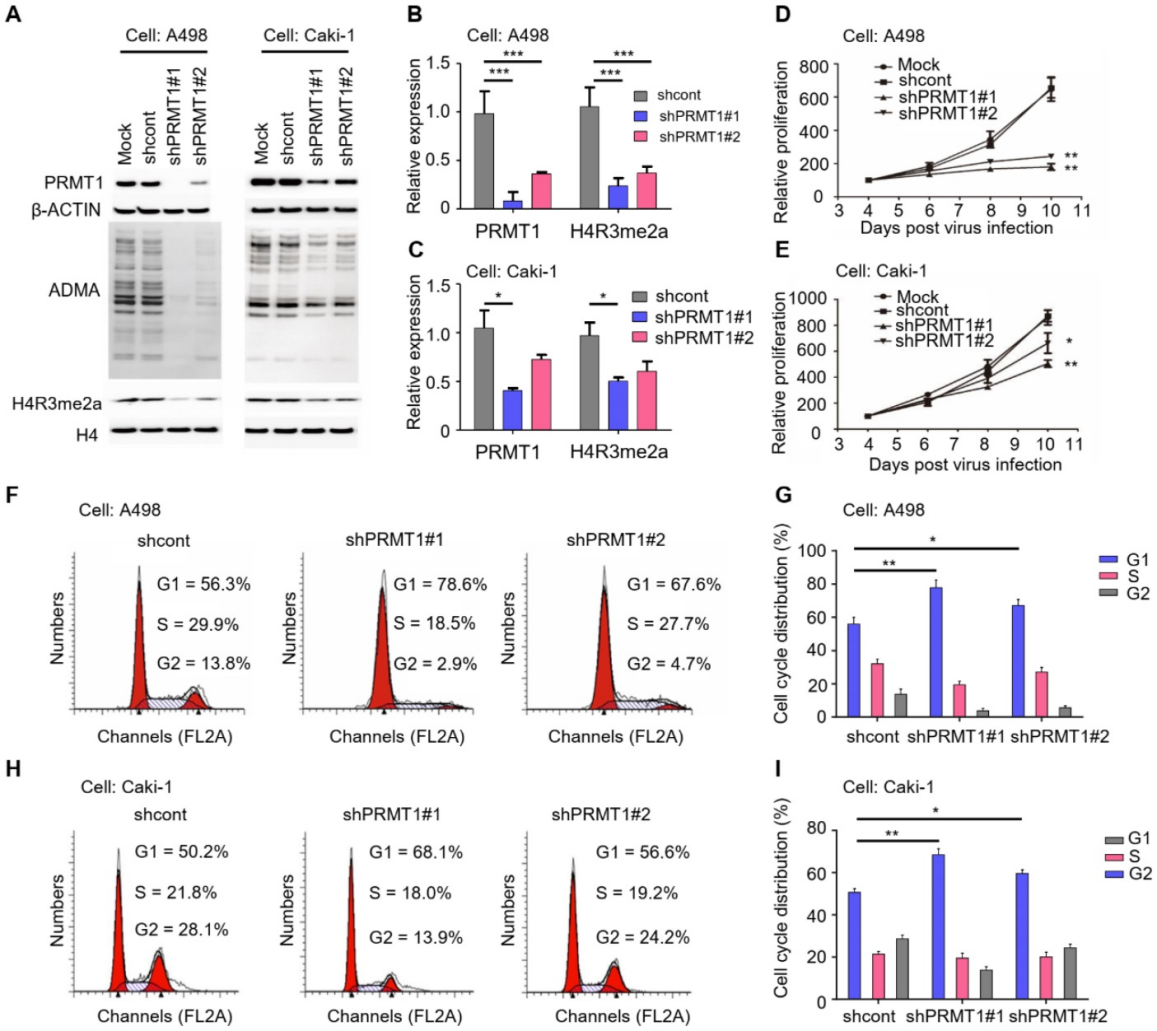
** Knockdown of PRMT1 suppresses the proliferation ability and induces G1 cell cycle arrest in ccRCC cells. (A-C)** Knockdown of PRMT1 in A498 and Caki-1 cells, and the expression of ADMA and H4R3me2a were detected by Western blotting. **(D-E)** SRB assay was performed to evaluate the proliferation of PRMT1-deleted A498 and Caki-1 cells. **(F-G)** Knockdown of PRMT1 significantly induced G1 cell cycle arrest in A498 cells; **(H-I)** Knockdown of PRMT1 significantly induced G1 cell cycle arrest in Caki-1 cells. *P < 0.05, **P < 0.01, and ***P < 0.001.

To elucidate the functional role of PRMT1 in ccRCC, we first knocked down PRMT1 expression in ccRCC cells (A498 and Caki-1). It has been reported that histone H4 arginine 3 asymmetric dimethylation (H4R3me2a) is the specific histone modification catalyzed by PRMT1 36. The knockdown efficiency was validated by Western blot which shows significantly decreased expression levels of PRMT1 as well as the ADMA and H4R3me2a levels after PRMT1 knockdown (**Figure 2A-C**; *P* < 0.05). We next evaluated the effect of PRMT1 knockdown on ccRCC cell growth, and results demonstrated that knockdown of PRMT1 significantly attenuated cell proliferation of ccRCC cells (**Figure 2D-E**; *P* < 0.05). In addition, an increased proportion in the G1 stage after PRMT1 knockdown was observed in ccRCC cells (**Figure 2F-I**). Taken together, our data suggested that PRMT1 plays a critical role in cellular proliferation and cell cycle progression of ccRCC cells.

### Targeted inhibition of PRMT1 activity induces G1 cell cycle arrest and suppresses proliferation of ccRCC cells

**Figure 3:**
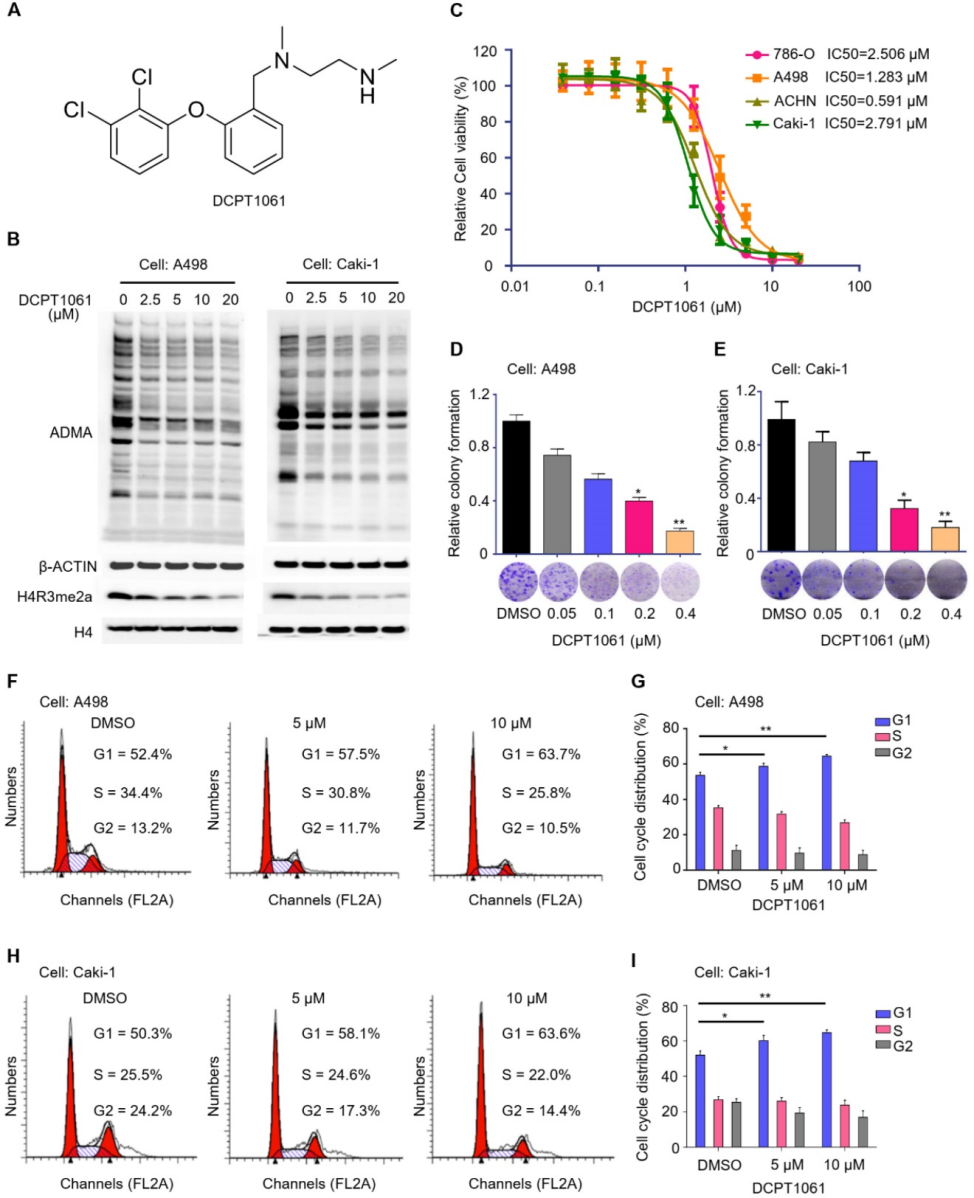
** Inhibition of PRMT1 activity suppresses cell growth and induces G1 cycle arrest in ccRCC cells. (A)** Structure of compound DCPT1061 with tartaric acid. **(B)** Treatment with compound DCPT1061 for 48 h dose-dependently inhibited the methyltransferase activity of PRMT1 in A498 and Caki-1 cells. ADMA, H4R3me2a, H3, and β-ACTIN expression levels were detected by Western blotting. **(C)** SRB assay was performed to determine the proliferation ability of ccRCC cells (786-O, A498, ACHN, Caki-1) treated with DCPT1061 for 7 days in a dose-dependent manner. **(D-E)** Colony formation of A498 and Caki-1 treated with different concentrations of DCPT1061. **(F-G)** Treatment with DCPT1061 for 48 h induced G1 cell cycle arrest in A498 cells.** (H-I)** Treatment with DCPT1061 for 48 h induced G1 cell cycle arrest in Caki-1 cells. *P < 0.05, **P < 0.01.

To test whether PRMT1 may serve as a potential therapeutic target for ccRCC treatment, we examined the effect of PRMT1 inhibitor on ccRCC cell proliferation. Through maintaining the side-chain structure (ethylenediamine chain) of our previously reported PRMT1 inhibitor while modifying different ring structures, we designed a new PRMT1 inhibitor DCPT1061 (**Figure 3A**). DCPT1061 potently inhibited PRMT1, PRMT6 and PRMT8* in vitro* with less inhibitory effect on PRMT3, PRMT4, and PRMT5 or other epigenetic enzymes (**Supplementary Figure S4**). DCPT1061 reduced cellular ADMA and PRMT1-mediated methylation mark H4R3me2a in a dose-dependent manner (**Figure 3B**), indicating the on-target inhibition of PRMT1 activity in cells. In four ccRCC cell lines including 786-O, A498, ACHN, and Caki-1, DCPT1061 significantly induced a dose-dependent inhibition of cell proliferation (**Figure 3C**), which is consistent with the effect of PRMT1 knockdown (**Figure 2D-E**). Colony formation assay further showed that DCPT1061 could suppress the clonogenic growth of ccRCC cells at a low concentration (**Figure 3D-E**). Additionally, as DCPT1061 is a new inhibitor of type I PRMTs, we also confirmed our results with another reported type I PRMTs inhibitor (GSK3368715) (**Supplementary Figure S5**). Furthermore, flow cytometry analysis demonstrated an increased percentage of cells in the G1 stage after DCPT1061 treatment in ccRCC cells (**Figure 3F-I**). Besides, we also determined whether PRMT1 inhibition inducing apoptosis in ccRCC cells, and the result showed that DCPT1061 barely impacts the apoptosis of ccRCC cells (**Supplementary Figure S6**). These results confirmed the therapeutic effect of DCPT1061 on ccRCC cells.

Because DCPT1061 treatment selectively inhibited PRMT1, PRMT6, and PRMT8 *in vitro*, we further determined whether DCPT1061 suppresses ccRCC cell proliferation through specific inhibition of PRMT1 or the other two. We found that knockdown of either PRMT6 or PRMT8 barely attenuated the growth of ccRCC cells (**Supplementary Figure S7A-D**), which is quite different from the anti-proliferative effects of PRMT1 knockdown (**Figure 2D-E**). Moreover, when either PRMT6 or PRMT8 was knocked down, DCPT1061 still potently inhibited ccRCC cell proliferation (**Supplementary Figure S7E-F**). On the contrary, DCPT1061 did not induce further growth inhibitory effects in PRMT1-deleted ccRCC cells (**Supplementary Figure S7G**), suggesting DCPT1061 suppressed the growth of ccRCC cells primarily through PRMT1, but not PRMT6 or PRMT8. Overall, targeting PRMT1 activity by DCPT1061 significantly inhibited ccRCC cell proliferation and induced G1 cell cycle arrest.

### Identification of LCN2 as a target gene of PRMT1

**Figure 4:**
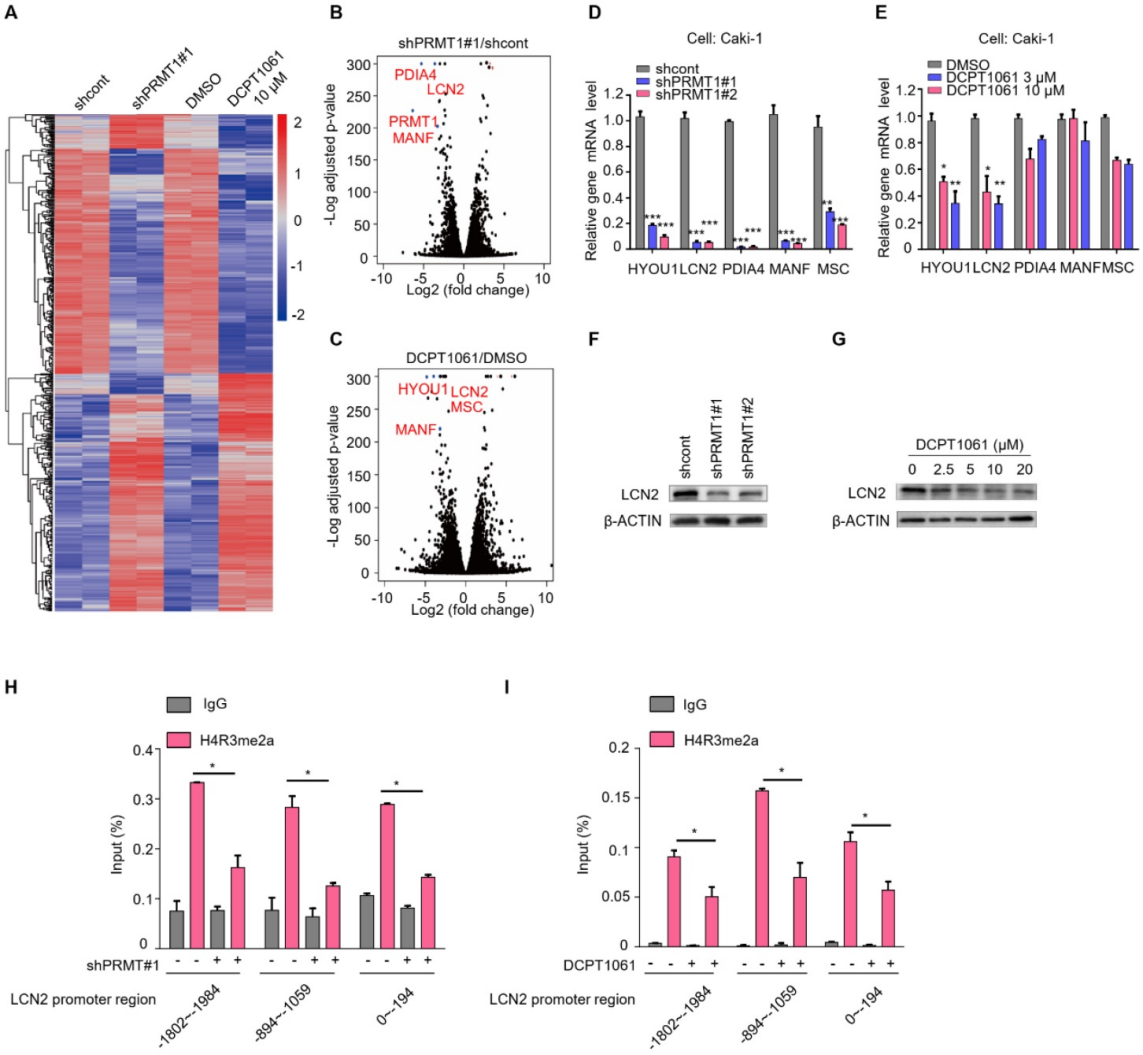
** Identification of LCN2 as a target gene of PRMT1. (A)** RNA-seq was performed to analyze gene expression changes in PRMT1 deleted or DCPT1061 treated Caki-1 cells. **(B-C)** The volcano plots were performed to show the significant differential expression genes in different groups. **(D-E)** Down-regulated genes were detected by RT-qPCR in PRMT1 deleted or DCPT1061 treated ccRCC cells. **(F-G)** LCN2 expression levels were detected by Western blot in PRMT1-deleted or DCPT1061 treated Caki-1 cells.** (H-I)** H4R3me2a binding at the three sections of *LCN2* promoter in PRMT1-deleted or DCPT1061 treated Caki-1 cells. *P < 0.05, **P < 0.01, and ***P < 0.001.

To investigate the mechanism underlying the anti-proliferative effects of DCPD1061 by targeting PRMT1, we firstly evaluated the relationship between PRMT1 and VHL-HIF pathway, which is the major genetic event in ccRCC. Results showed that PRMT1 inhibition shows no significant correlation with HIF-1a expression and we believe there is no directed relation between PRMT1 and VHL-HIF in ccRCC (**Supplementary Figure S8**). Furthermore, RNA-sequencing (RNA-seq) analysis was performed to analyze gene expression changes in ccRCC cells that were treated with shPRMT1 lentivirus or DCPT1061 (**Figure 4A**). Volcano plots analysis revealed the top differential genes which were most significantly regulated by PRMT1 knockdown and chemical inhibition (**Figure 4B-C**). RT-qPCR confirmed the reduced transcription of these genes, among which *HYOU1* and *LCN2* are the most significantly inhibited by PRMT1 knockdown or DCPT1061 treatment (**Figure 4D-E**). Further, LCN2 knockdown turned out to show the most potent inhibition on cell proliferation when we examined the roles of these genes on ccRCC cell proliferation through knockdown experiments. Western botting analysis confirmed that LCN2 expression was inhibited by both PRMT1 knockdown and its inhibition by DCPT1061 in ccRCC cells (**Figure 4F-G**).

Furthermore, we investigated the detailed molecular mechanism underlying how PRMT1 regulates LCN2 expression in ccRCC cells. H4R3me2a, which is the specific histone modification catalyzed by PRMT1, has always been considered as a transcriptional active marker for gene expression 37. We performed the chromatin immunoprecipitation ChIP-qPCR assay in PRMT1 deleted or inhibited ccRCC cells. Results showed that H4R3me2a mark bound to the promoter region of *LCN2* gene. In three promoter regions of *LCN2*, the enriched level of H4R3me2a was significantly decreased after PRMT1 knockdown or inhibition (**Figure 4H-I**). Taken together, these results demonstrate that PRMT1 deficiency could regulate the level of H4R3me2a on *LCN2* promoter to repress its expression.

### LCN2 is a functional mediator of PRMT1 in ccRCC cells

**Figure 5:**
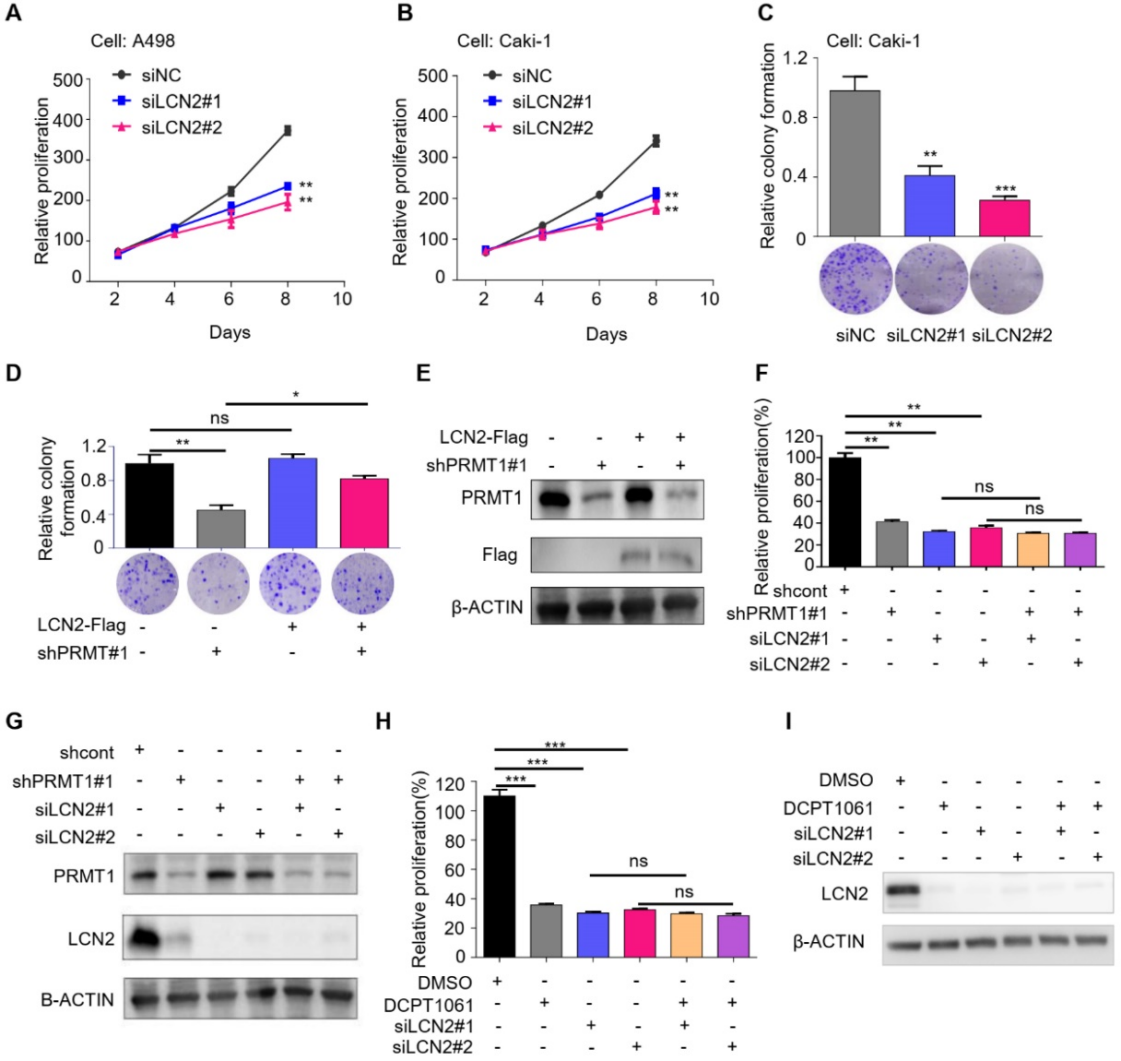
** LCN2 is a functional mediator of PRMT1 in ccRCC cells. (A-B)** SRB assays were performed to evaluate the proliferation of LCN2-deleted A498 and Caki-1 cells.** (C)** Colony formation ability in LCN2-deleted Caki-1 cells.** (D-E)** Colony formation assay was performed to detect cell colony formation ability of restoring the expression of LCN2 in PRMT1-deleted Caki-1 cells.** (F-G)** SRB assay was performed to detect cell proliferation of knocking down of PRMT1 in LCN2-deleted Caki-1 cells. **(H-I)** SRB assay was performed to detect cell proliferation of DCPT1061 treated and/or LCN2 deleted Caki-1 cells. *P < 0.05, **P < 0.01, and ***P < 0.001.

LCN2 has been shown to play important roles in tumor cell survival, proliferation, and metastasis, and its increased expression levels correlate with higher histological grade and a worse prognosis in various human malignancies, including breast cancer, ovarian carcinoma, pancreatic cancer, gastric cancer, and thyroid carcinoma 27, 38-41. We showed that LCN2 is also a crucial regulator of ccRCC cell proliferation by assessing the effects of LCN2 knockdown on ccRCC cells. Results showed that knockdown of LCN2 significantly impaired cell proliferation and clonogenic growth (**Figure 5A-C**). In addition, flow cytometry analysis indicated that knockdown of LCN2 led to G1 cell cycle arrest, which was coherent to the phenotype caused by PRMT1 deficiency (**Supplementary Figure S9**).

We further investigated whether the inhibitory effects of PRMT1 deficiency on cell proliferation were mediated by the suppression of LCN2. We performed a rescue experiment and found that restored expression of LCN2 rescued the suppression of colony formation caused by PRMT1 deficiency (**Figure 5D-E**). Moreover, PRMT1 knockdown or inhibition suppressed cell growth in control cells but did not induce further growth inhibition in LCN2-deleted cells (**Figure 5F-I**). These results suggested that PRMT1 deficiency inhibited ccRCC cell proliferation through downregulating LCN2 expression, presenting LCN2 as a critical functional mediator of PRMT1 in regulating ccRCC cell proliferation.

### The AKT pathway is involved in PRMT1-regulated LCN2 expression in ccRCC cells

**Figure 6:**
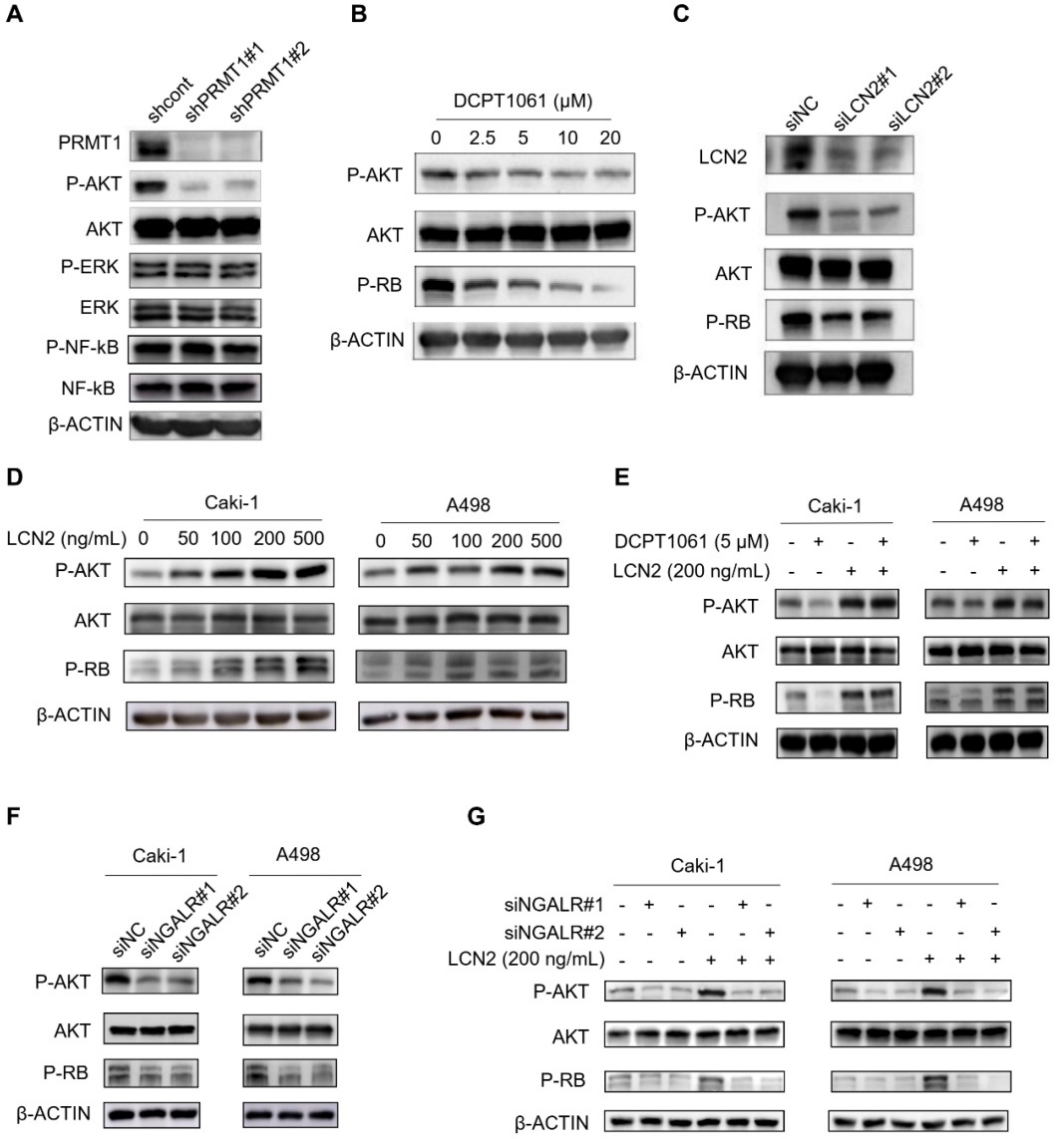
**The AKT pathway is involved in PRMT1-regulated LCN2 expression in ccRCC cells. (A)** The AKT pathway was inactivated after PRMT1 knockdown in Caki-1 cells, but no effect on the ERK1/2 and NF-κB pathways was observed. **(B)** DCPT1061 inhibited AKT and RB phosphorylation in Caki-1 cells. **(C)** Knockdown of LCN2 inhibited AKT and RB phosphorylation in Caki-1 cells.** (D)** Exogenous LCN2 stimulation at different concentrations induced AKT-RB activation in Caki-1 and A498 cells.** (E)** Exogenous LCN2 stimulation (200 ng/ml) reactivated the AKT-RB pathway in DCPT1061 treated Caki-1 cells. **(F)** The AKT-RB pathway was inactivated after NGALR siRNAs knockdown in Caki-1 and A498 cells, as compared with the scrambled control. **(G)** Exogenous LCN2 stimulation (200 ng/mL) barely activated the AKT-RB pathway in NGALR-deleted Caki-1 cells.

Based on our above findings, we next explored the pathways involved in PRMT1-regulated LCN2 expression associated with ccRCC cell proliferation. An increasing number of signal pathways, such as AKT, ERK1/2 and NF-κB signal pathways, are consecutively reported to play important roles in PRMT1 oncogenic functions in cancers 42-45. As shown in **Figure 6A**, we found the AKT pathway was significantly inactivated following PRMT1 knockdown in ccRCC cells, whereas the ERK1/2 and NF-κB pathways were not affected. Furthermore, the AKT-RB pathway was also inactivated by PRMT1 enzymatic inhibition or LCN2 genetic deletion in ccRCC cells (**Figure 6B-C**). Besides, PRMT1 inhibition by GSK3368715 also confirmed this result (**Supplementary Figure S10**). Given that the expression level of LCN2 is transcriptionally regulated by PRMT1, we next examined the role of exogenous LCN2 protein and found that LCN2 could activate the AKT-RB pathway in ccRCC cells (**Figure 6D**). Moreover, we next want to explore whether exogenous LCN2 protein could rescue the suppression of the AKT-RB pathway induced by PRMT1 inhibition in ccRCC cells, and revealed that the exogenous LCN2 protein could significantly reactivate the AKT-RB pathway in DCPT1061 treated ccRCC cells (**Figure 6E**). Our findings suggest that the expression of LCN2 regulated by PRMT1 may contribute to the activation of the AKT-RB signal pathway in ccRCC.

It is reported that LCN2, a glycoprotein involved in multiple biological processes, functions through the NGAL receptor (NGALR) 46. To evaluate whether NGALR is involved in LCN2-mediated regulation of the AKT-RB signal pathway, NGALR was genetically depleted by siRNAs in ccRCC cells. Results showed that knockdown of NGALR could significantly suppress the AKT-RB signal pathway, whereas adding exogenous LCN2 protein barely rescued the suppression of the AKT-RB pathway induced by NGALR genetic deletion in ccRCC cells (**Figure 6F-G**). Taken together, our data indicated that PRMT1-regulated LCN2 expression that activates the AKT-RB pathway, which may responsible for the proliferation of ccRCC cells.

### Combinational treatment of DCPT1061 and sunitinib exhibited a striking anticancer effect in ccRCC

**Figure 7:**
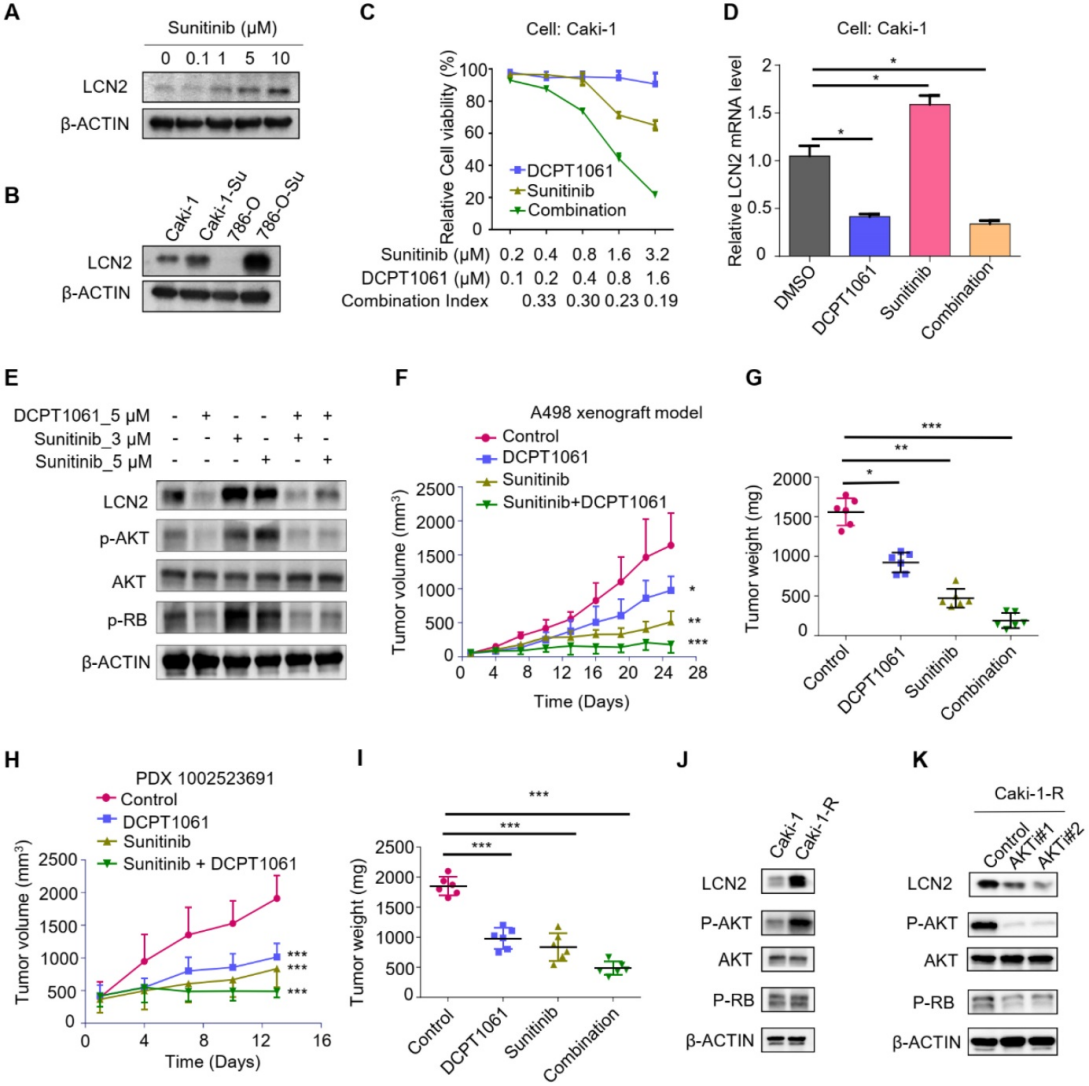
** Combinational treatment of DCPT1061 and sunitinib exhibited a striking anticancer effect in ccRCC. (A)** LCN2 and β-ACTIN protein levels were detected by Western blotting in sunitinib treated Caki-1 cells. **(B)** LCN2 and β-ACTIN protein levels were detected by Western blotting in Caki-1 cells, sunitinib long exposed Caki-1 (Caki-1-Su) cells, 786-O cells, and sunitinib long exposed 786-O (786-O-Su) cells. **(C)** Cell proliferation of Caki-1 cells was evaluated following 72 h exposure to the indicated compounds. Combination index (CI) was used to determine the synergistic effect of sunitinib and DCPT1061 treatment.** (D)** LCN2 mRNA levels were detected by RT-qPCR in sunitinib and/or DCPT1061 treated Caki-1 cells.** (E)** LCN2, p-AKT, AKT, p-RB, and β-ACTIN protein levels were detected by Western blotting in sunitinib and/or DCPT1061 treated Caki-1 cells. **(F-G)** Tumor growth curves in A498 xenograft models, and tumor weights were expressed as mean ± SD, n = 6. **(H-I)** Tumor growth curves in PDX (1002523691) xenograft models, and tumor weights were expressed as mean ± SD, n = 6. **(J)** LCN2, p-AKT, AKT, p-RB, and β-ACTIN protein levels were detected by Western blotting in sunitinib resistant Caki-1 cells. **(K)** LCN2 protein levels were detected by Western blotting in AKT inhibitor (AKTi#1, GSK 2110183; AKTi#2, BKM120) treated Caki-1 cells. *P < 0.05, **P < 0.01, and ***P < 0.001.

Poised epigenetic states in tumor cells have been reported to be associated with drug resistance, which suggests it is probable to return to a drug-sensitive state if the epigenetic effect is prohibited 47. We noticed that sunitinib treatment upregulated the expression of LCN2, and the increase of LCN2 expression was much more obvious in ccRCC cells which were exploded to long-term sunitinib treatment (**Figure 7A-B**). It has been reported that LCN2 was involved in sunitinib resistance 48, 49, we thus tested whether DCPT1061-mediated LCN2 suppression could sensitize ccRCC to sunitinib treatment. A significant synergistic anti-proliferative effect was observed when ccRCC cells were treated with DCPT1061 and sunitinib in combination (**Figure 7C and Supplementary Figure S11A**). Western blotting analysis confirmed that the expression of LCN2, as well as its downstream p-AKT and p-RB levels, was increased upon sunitinib treatment, which was abrogated by PRMT1 inhibition in the combination group (**Figure 7D-E**). These data indicated that DCPT1061 reduced the upregulation of the LCN2-AKT-RB axis caused by sunitinib, and sensitized ccRCC to sunitinib treatment.

To further determine the therapeutic effect of DCPT1061 as well as its combination with sunitinib treatment for ccRCC *in vivo*, we first constructed two ccRCC xenograft animal models using A498 and Caki-1 cell lines. We found that the single treatment with DCPT1061 or sunitinib showed certain effects on ccRCC, while the combination of DCPT1061 and sunitinib treatment remarkably suppressed the tumor growth (**Figure 7F-G and Supplementary Figure S11B-C**; *P* < 0.05). Besides, we adopted a PDX model to further confirm the efficacy of DCPT1061 and the combination regimen. We demonstrated DCPT1061 or sunitinib alone significantly inhibited the PDX tumor growth, while the combination therapy of DCPT1061 and sunitinib showed much more efficiency (**Figure 7H-I**, *P* < 0.05). Furthermore, we also performed a drug withdrawal experiment in the Caki-1 xenograft animal model. Results showed that tumor growth revived in the DCPT1061 or sunitinib treatment group while exhibited constant remission in the combination group after the withdrawal of drug treatment (**Supplementary Figure S11D-E**; *P* < 0.05). Notably, in terms of tolerance and toxicity, no significant weight loss or hematological changes were observed in either group of mice (**Supplementary Figure S11F-I and Table S6**). We further performed the IHC analysis of LCN2 expression levels in tumor tissues. Results showed an increased expression level of LCN2 in the sunitinib treated group compared with the control group, while a decreased LCN2 expression level was detected in the DCPT1061 treated group and in the combination group (**Supplementary Figure S12**). In addition, the expression of p-AKT and p-RB were in line with the LCN2 expression profile (**Supplementary Figure S12**).

Next, we considered the possibility that sunitinib-induced increase in LCN2 expression is mediated by AKT in a feed-forward loop. Thus, we tried to construct a sunitinib-resistant ccRCC cell line (Caki-1-R) cell line and found that the AKT pathway was activated in Caki-1-R cells (**Figure 7J and Supplementary Figure S13**). Further, we examined whether inhibition of the AKT pathway using chemical inhibitors would block the increase expression of LCN2 in sunitinib-resistant ccRCC cells. Indeed, we revealed that LCN2 expression was strikingly suppressed in Caki-1-R cells treated with AKT inhibitors, indicating there is a feed-forward loop between LCN2 expression and the AKT pathway in sunitinib-resistant ccRCC cells (**Figure 7K**). Considering the reported mechanisms of sunitinib resistance, such as increased production of IL-8 and upregulation of c-MET, we next explored the correlation between the PRMT1 and IL-8 or c-MET expression in Caki-1-R cells. Results showed PRMT1 inhibition barely impacted the expression of IL-8 and c-MET in Caki-1-R cells (**Supplementary Figure S14**). Thus, we excluded these potential interferences and made our conclusion appropriate. In summary, treatment of PRMT1 inhibitor DCPT1061 reversed the upregulation of LCN2-AKT-RB by sunitinib and sensitized ccRCC to sunitinib treatment* in vitro* and* in vivo*.

## Discussion

Currently, the exact mechanism of ccRCC development remains elusive, and treatment for advanced ccRCC is mainly limited to targeted therapies and immunotherapies, which are facing many challenges 50. Aberration in epigenetic regulators is reported to be involved in tumor progression and drug resistance. Arginine methylation is a prevalent but reversible protein modification that regulates important biological processes including gene transcription and signal transduction 12. PRMT1 is the primary type I enzyme of protein arginine methyltransferase family that accounts for ~90% of cellular arginine methylation events. In addition, an increasing body of evidence suggests that PRMT1 plays a crucial role in cancers. Aberrant expression of PRMT1 was identified in hepatocellular carcinoma, chondrosarcoma, MLL-rearranged acute lymphoblastic leukemia, pancreatic cancer, colon cancer, and many other tumors, which was highly related to cancer progression and prognosis 14, 16, 51-54. However, whether PRMT1 plays a role in the genesis and progression of ccRCC is yet to be elucidated. In this study, we reported that PRMT1 is significantly upregulated in ccRCC tissues and correlated with poor clinical features of ccRCC patients. We further constructed prognostic nomograms for ccRCC survival analysis and demonstrated PRMT1 expression level, when combined with several conventional clinical features, may help to predict the outcomes of ccRCC patients with high accuracy. Interestingly, the results of our study contradict a previous study on PRMT1 in ccRCC. Filipovic et al. reported that PRMT1 expression level was decreased in ccRCC tissues, and its expression level was associated with a better cancer prognosis of ccRCC patients 18. It is unclear why there is a difference between these two studies. Racial differences might account for this contradiction. Our analysis included patients who are all Chinese people, whereas there were most European in the previous study. This phenomenon has also been reported in other tumors. Williams DJ et al. reported that the biological characteristics and prognosis of triple-negative breast cancer have obvious diversity between populations 55. Additionally, the different amounts of ccRCC patients in each cohort may influence the results. In our cohort, 358 ccRCC patients were analyzed, whereas 120 ccRCC samples were included in the previous study. In addition, the length of time for ccRCC patient's follow-up might also impact the results. The median follow-up time in our cohort was 106.4 months, whereas 44.2 months in the previous study. Therefore, our data might not be entirely persuasive to illustrate the expression profile of PRMT1 in ccRCC and more efforts are needed to research this problem. Regardless, our findings suggest that increased PRMT1 may participate in the development of ccRCC and PRMT1 expression level may serve as a novel prognostic marker for ccRCC patients.

By using a combinatory approach of genetic manipulation and chemical intervention, we showed PRMT1 could serve as a drug target in ccRCC. Genetic knockdown of PRMT1, as well as DCPT1061 intervention, consistently attenuated the proliferation and induced G1 cell cycle arrest of ccRCC cells, indicating the growth inhibition induced by DCPT1061 treatment was due to the on-target inhibition. Mechanistically, we showed PRMT1 knockdown or pharmacological inhibition transcriptionally reduced LCN2 expression through the regulation of H4R3me2a deposited at its promoter region, leading to reduced AKT-RB signaling. LCN2 has been reported to play important roles in various human malignancies, including breast cancer, ovarian carcinoma, pancreatic cancer, gastric cancer, and thyroid carcinoma 27, 38-41. LCN2 was identified as an oncogene that drives tumor progression in papillary renal cell carcinoma 56. Upregulated serum LCN2 level was also reported in ccRCC, which correlated with a higher histological grade and a worse prognosis of ccRCC 57. In our study, we tested the effects of LCN2 knockdown on ccRCC cell proliferation, indicating that LCN2 was also crucial for ccRCC proliferation. Moreover, restored the expression of LCN2 rescued cell proliferation inhibited by PRMT1 deficiency, while LCN2 knockdown abrogated the anti-proliferative effects of DCPT1061. Together, these results suggested that LCN2 functions as a downstream effector of PRMT1 and DCPT1061 treatment inhibited ccRCC growth at least partially through the reduction of LCN2 expression and the downstream AKT-RB pathway.

Though targeted therapy and immunotherapy bring new hopes, advanced ccRCC patients still face tremendous therapeutic challenges such as low response rates and notorious drug resistance 50. Epigenetic medicines are emerging as novel treatments in drug combination which may help prevent or overcome drug resistance 58.In the current study, strong synergistic anti-proliferative effects were observed between DCPT1061 and sunitinib treatment *in vitro*. We also found DCPT1061 suppressed the tumor growth of ccRCC alone and further sensitize ccRCC to sunitinib treatment in ccRCC CDX models as well as in the PDX model. More importantly, when we terminated the drug dosing after a two-week treatment, the combination group continued to display a significant tumor remission, supporting a durable effect upon the combination regimen. LCN2 has previously been reported to be involved in sunitinib resistance in ccRCC, and has also been implicated in the resistance of another TKI inhibitor sorafenib, an approved drug for the treatment of liver cancer 59. In this study, we found that sunitinib treatment induced upregulation of LCN2, the crucial mediator of sunitinib resistance. The increase of LCN2 expression, as well as its downstream AKT-RB signaling by sunitinib, was nearly blocked by DCPT1061 treatment *in vitro*, explaining the mechanism underlying the synergism between sunitinib and DCPT1061. Western blot and IHC analysis of the tumor tissues from the animal experiment also confirmed that sunitinib treatment increased LCN2 expression and downstream AKT-RB level, and this signaling pathway was significantly reduced by DCPT1061 treatment in the combination group. These data together indicated downregulation of LCN2-AKT-RB by DCPT1061 could potentially overcome sunitinib-resistance in ccRCC, supporting that DCPT1061 alone or in combination with sunitinib, might serve as promising effective therapeutic strategies for advanced ccRCC treatment.

PRMT1 has been reported to play important roles in tumor progression, yet whether PRMT1 may serve as a therapeutic target for cancer treatment requires assessing the therapeutic effects of targeting PRMT1 pharmacologically. Various PRMT1 inhibitors have been developed in recent years. So far, the biochemical potency of known PRMT1 selective inhibitors have been almost trapped in micromoles, while most of the reported PRMT1 inhibitors lack the assessment from cellular and animal levels 60. Our recently reported PRMT1 inhibitor, DCPR049_12 61, potently inhibited PRMT1 enzymatic activity at the nanomolar level *in vitro*, yet they also inhibited other members in type I PRMTs including PRMT3, PRMT4, PRMT6, and PRMT8. Its cellular activity was much lower compared to its* in vitro* inhibitory activity, probably due to low cell permeability, which required further chemical optimization. Based on DCPR049_12, we designed DCPT1061 with improved selectivity among type I PRMT subfamily members. Although DCP1061 potently inhibited PRMT1, PRMT6, and PRMT8 *in vitro*, cellular experiments confirmed that DCPT1061 inhibited ccRCC cell proliferation through inhibition of PRMT1, instead of PRMT6 or PRMT8. The use of DCPT1061 as a chemical tool helped us achieve a better understanding of PRMT1 function in ccRCC and present PRMT1 as a druggable target for the treatment of ccRCC.

## Conclusions

Taken together, we identified PRMT1 as a critical regulator of ccRCC progression. Targeting PRMT1 with DCPT1061, a novel PRMT1 inhibitor, showed pharmacological efficacy both *in vitro* and *in vivo* and sensitized ccRCC to the first-line drug sunitinib. Therefore, pharmacological inhibition of PRMT1 by DCPT1061 might represent as an effective therapeutic approach for the treatment of advanced ccRCC. Mechanistically, PRMT1 knockdown as well as pharmacological inhibition transcriptionally inhibited LCN2 expression, thereby inhibiting the downstream AKT-RB pathway and ultimately attenuating the proliferation of ccRCC as well as sensitizing ccRCC to sunitinib treatment. Given the vital functional role of PRMT1 in ccRCC, strategies targeting PRMT1 may fulfill the unmet medical needs as promising clinical therapies for advanced ccRCC.

## Supplementary Material

Supplementary figures and tables.

## Figures and Tables

**Table 1:** Association of PRMT1 expression with clinicopathological characteristics in 358 ccRCC patients

Characteristics	Patients		Tumoral PRMT1 expression		
	n	%	Low	High	*P*
All patients	358	100	119	239	
**Gender**					0.698
Male	254	70.9	86	168	
Female	104	29.1	33	71	
**Age (years)**					0.681
≤ 55	178	49.7	61	117	
> 55	180	50.3	58	122	
**TNM stage**					0.122
I+II	336	93.9	115	211	
III+IV	22	6.1	4	18	
**Invasion depth (T stage)**					0.402
T1+T2	344	96.1	116	228	
T3+T4	14	3.9	3	11	
**Lymph nodes metastasis (N stage)**					0.032*
N0	349	97.5	119	230	
N1	9	2.5	0	9	
**Distant metastasis (M stage)**					0.668
M0	352	98.3	118	234	
M1	6	1.7	1	5	
**Pathological grade**					0.027*
I+II	293	81.8	105	188	
III+IV	65	18.2	14	51	
**Tumor size (cm)**					0.682
≤ 4	186	52	60	126	
> 5	172	48	59	113	

*P < 0.05 indicates a significant association among the variables.

**Table 2:** Multivariate cox regression analysis for overall survival and recurrence-free survival in ccRCC patients

Variables	Overall survival			Recurrence-free survival		
	HR	(95% CI)	*P*	HR	(95% CI)	*P*
**PRMT1 in cancer tissues**						
Low	1			1		
High	2.493	1.337-4.648	0.004*	2.735	1.453-5.151	0.002*
**Age (years)**						
≤55						
>55	1.389	0.840-2.297	0.200			
**Clinical stage**						
I+II	1			1		
III+IV	1.474	0.492-4.413	0.488	1.137	0.391-3.304	0.813
**Invasion depth (T stage)**						
T1+T2	1			1		
T3+T4	2.735	0.903-8.280	0.075	2.065	0.693-6.149	0.193
**Lymph nodes metastasis (N stage)**						
N0	1			1		
N1	3.723	1.349-10.278	0.011*	3.941	1.341-11.587	0.013*
**Distant metastasis (M stage)**						
M0	1			1		
M1	1.601	0.544-4.715	0.393	1.525	0.462-5.027	0.488
**Pathological grade**						
I+II	1			1		
III+IV	2.277	1.374-3.775	0.001*	2.104	1.275-3.473	0.004*
**Tumor size (cm)**						
≤4	1			1		
>4	3.867	2.055-7.275	<0.001*	5.227	2.751-9.929	<0.001*

HR: hazard ratio, 95% CI: 95% confidence interval, *P < 0.05 was considered statistically significant.

**Table 3:** Comparison of the predictive accuracy of prognostic factors

Model	Overall Survival (N=358)		Recurrence free survival (N=358)	
	C-Index	AIC	C-Index	AIC
PRMT1	0.606	795.4821	0.6	829.4477
N stage	0.563	777.9406	0.55	819.1828
N stage+ PRMT1	0.646	770.5319	0.632	810.3368
Pathological grade	0.627	783.0551	0.624	820.6104
Pathological grade+ PRMT1	0.683	775.3126	0.686	811.8178
Tumor size	0.683	766.2329	0.703	793.0312
Tumor size+ PRMT1	0.736	753.141	0.754	777.5203
Nomogram	0.771	723.6522	0.788	757.2218

AIC: Akaike information criterion C-index: Harrell's concordance index.

## Abbreviations

PRMT1: protein arginine methyltransferase 1
ccRCC: clear cell renal cell carcinoma
LCN2: lipocalin2
TKIs: tyrosine kinase inhibitors
MMA: ω-NG-monomethylation
ADMA: NG-asymmetric dimethylation
SDMA: NG′-symmetric dimethylation
TMAs: tissue microarrays
PDX: patient-derived tumor xenograft
GENT: Gene Expression database of Normal and Tumor tissues
TCGA: The Cancer Genome Atlas
RT-qPCR: quantitative reverse transcription polymerase chain reaction
OS: overall survival
RFS: recurrence-free survival
CETSA: cellular thermal shift assay
ITDRF: isothermal dose-response fingerprint
IHC: immunohistochemistry
PI: propidium iodide
