# Supplementary material for: PRMT1 is a novel molecular therapeutic target for clear cell renal cell carcinoma
Source: Theranostics. 2021 Mar 12;11(11):5387–403. doi: 10.7150/thno.42345 (PMC8039964; doi:10.7150/thno.42345)
Supplement: Supplementary file 1 — Supplementary figures and tables. [file thnov11p5387s1.pdf]

Supplementary data

Enzymatic activity measurement.

The selectivity of DCPT1061 was determined against 17 different kinds of epigenetic targets as described previously (1). The selectivity against enzymes of the PRMT family including PRMT1, PRMT3, CARM1 (PRMT4), PRMT5, PRMT6, and PRMT8 and a panel of other targets including DNMT1, DNMT3A/3L, DOT1L, EZH2, G9a, GCN5, LSD1, MLL1, PRDM9, SMYD2, SUV39H1 was measured in Shanghai Chempartner Co.Ltd through radioisotope assay or the AlphaLISA assay.

Cell apoptosis analysis

Apoptosis of ccRCC cells was detected by flow cytometry. Cells were stained with recombinant FITC-conjugated annexin V and propidium iodide (Becton-Dickinson, New Jersey, USA) according to the manufacturer's instructions.

Reference

1. Wang C, Jiang H, Jin J, Xie Y, Chen Z, Zhang H, et al. Development of Potent Type I Protein Arginine Methyltransferase (PRMT) Inhibitors of Leukemia Cell Proliferation. J Med Chem. 2017; 60(21): 8888-905.

Table S1 RT-qPCR primer sequences.

Gene	Forward	Reverse
PRMT1	CTTTGACTCCTACGCACACTT	GTGCCGGTTATGAAACATGGA
PRMT6	TACCGCCTGGGTATCCTTCG	CCTGTTCCGGCAACTCTACA
PRMT8	CCTGCTAAGCCCGTGCAAT	TGGGCATAGGAGTCGAAGTAA
LCN2	CCACCTCAGACCTGATCCCA	CCCCTGGAATTGGTTGTCCTG
NGALR	GTTACCCCGCAGACAGATTTG	CCCAAGAGGAATCGGAGGG
HYOU1	GAGGAGGCGAGTCTGTTGG	GCACTCCAGGTTTGACAATGG
PDIA4	GGCAGGCTGTAGACTACGAG	TTGGTCAACACAAGCGTGACT
MANF	TTTACCAGGACCTCAAAGACAGA	TTGCTTCCCGGCAGAACTTTA
MSC	CCCCGACACTAAGCTCTCCA	GTAGCCGTTCTCATAGCGGT
IL-8	TTTTGCCAAGGAGTGCTAAAGA	AACCCTCTGCACCCAGTTTTC
c-MET	AGCAATGGGGAGTGTAAAGAGG	CCCAGTCTTGTACTCAGCAAC
β-ACTIN	GAGACCTTCAACACCCCAGC	ATGTCACGCACGATTTCCC

Table S2 Catalog of antibodies and their applications.

Target protein	Antibody	Application
PRMT1	#2449 (Cell Signaling Technology)	WB
PRMT1	ab190892 (Abcam)	IHC
β-actin	60008-1-Ig (Proteintech)	WB
ADMA	#13522 (Cell Signaling Technology)	WB
H4R3me2a	#39705 (Active Motif)	WB/ChIP
H4R3me2a	ab194683 (Abcam)	IHC
H4	ab10158 (Abcam)	WB
LCN2	ab125075 (Abcam)	WB
LCN2	ab41105 (Abcam)	IHC
p-AKT	#4060 (Cell Signaling Technology)	WB/IHC
AKT	#4691 (Cell Signaling Technology)	WB
p-ERK	#4370 (Cell Signaling Technology)	WB
ERK	#4695 (Cell Signaling Technology)	WB
P-NF-kB	#3033 (Cell Signaling Technology)	WB
NF-kB	#8242 (Cell Signaling Technology)	WB
HIF-1A	#36169 (Cell Signaling Technology)	WB
p-RB	#8516 (Cell Signaling Technology)	WB/IHC

Table S3 Sequence-specific siRNAs for genes silence.

Gene	Forward	Reverse
siNC	UUCUCCGAACGUGUCACGUTT	ACGUGACACGUUCGGAGAATT
siLCN2#1	CCACCAUCUAUGAGCUGAATT	UUCAGCUCAUAGAUGGUGGTT
siLCN2#2	CAGCAUGCUAUGGUGUUCUTT	AGAACACCAUAGCAUGCUGTT
siNGALR#1	CCGAUUCCUCUUGGGCUUUTT	AAAGCCCAAGAGGAAUCGGTT
siNGALR#2	GCCCUUGUCUCUAAGGAUUTT	AAUCCUUAGAGACAAGGGCTT
siPRMT6#1	CCGGCACCGGCATTCTGAGCATCTTCTCGA	GAAGATGCTCAGAATGCCGGTGTTTTTG
siPRMT6#2	GCACCGGCAUUCUGAGCAUTT	AUGCUCAGAAUGCCGGUGCTT
siPRMT8#1	CCAUGUACCACAACAAGCA	UGCUUGUUGUGGUACAUGG
siPRMT8#2	GACCUAGGUGUUCUCUCAG	CUGAGAGAACACCUAGGUC

Table S4. Primer sequences for LCN2 promoter.

Gene promoter	Forward	Reverse
LCN2_Promoter_1	tggagtgcaatggcgcaaccttg	caggtgttcaagaccagcctgac
LCN2_Promoter_2	ctctaaaaccagcgaaccctcag	cagcagggaagcagttatctcaa
LCN2_Promoter_3	cacagagggagtcgttgtccctg	atttatgggatctagggtgggttg

Table S5. The expression pattern of PRMT1 in ccRCC and paired normal tissues.

Tissue sample	No. of patients	Expression of PRMT1 (n, %)		*P*-Value
		High	Low	
Tumor	358	239 (66.8%)	119 (33.2%)	<0.001*
Normal	234	90 (38.5%)	144 (61.5%)	

*P<0.05 was considered statistically significant.

Table S6. BALB/c-nu/nu mice hematology.

Test item (units)	Control	11	Sunitinib	Combination	Reference range
WBC (10^3/μl)	5.7±2.9	6.8±1.4	7±1.6	6±2.3	0.8-6.8
Lymph (10^3/μl)	3.1±1.0	2.7±1	3.2±0.9	2.8±1.4	0.7-5.7
Mono (10^3/μl)	0.5±0.3	0.4±0.3	0.4±0.2	0.3±0.1	0.0-0.3
Gran (10^3/μl)	2.9±0.9	3.7±1.4	3.4±1.1	2.9±0.9	0.1-1.8
Lymph (%)	47.7±5.1	39.8±14.2	45.6±9.3	46±5.2	55.8-90.6
Mono (%)	6.7±2.8	5.9±3.6	6±2.4	4.8±1.3	1.8-6.0
Gran (%)	45.7±5.1	54.3±15.7	48.4±8.1	49.2±5.5	8.6-38.9
RBC (10^6/μl)	10.2±2.2	11.2±0.7	10.5±1.2	10.5±0.8	6.36-9.42
HGB (g/l)	163.3±37.3	183.3±13.1	174.1±21.3	169.6±12.6	110-143
HCT (%)	52.3±11.6	57.5±4.2	55.1±6.5	55.3±3.3	34.6-44.6
MCV (fl)	51.1±0.7	51.2±0.7	52.6±0.8	52.6±1.4	48.2-58.3
MCH (pg)	15.9±0.3	16.3±0.3	16.6±0.3	16.1±0.8	15.8-19
MCHC (g/l)	311.3±5.8	318.3±3.6	315.4±3.3	306.1±10.6	302-353
RDW (%)	14.4±0.5	14.3±0.5	16.3±0.7	16±0.9	13-17
PLT (10^3/μl)	788.9±354.4	992±274.8	1124.4±198.7	1043.9±555.9	450-1590
MPV (fl)	6.1±0.4	6.4±0.5	6.3±0.4	6.7±0.9	3.8-6.0

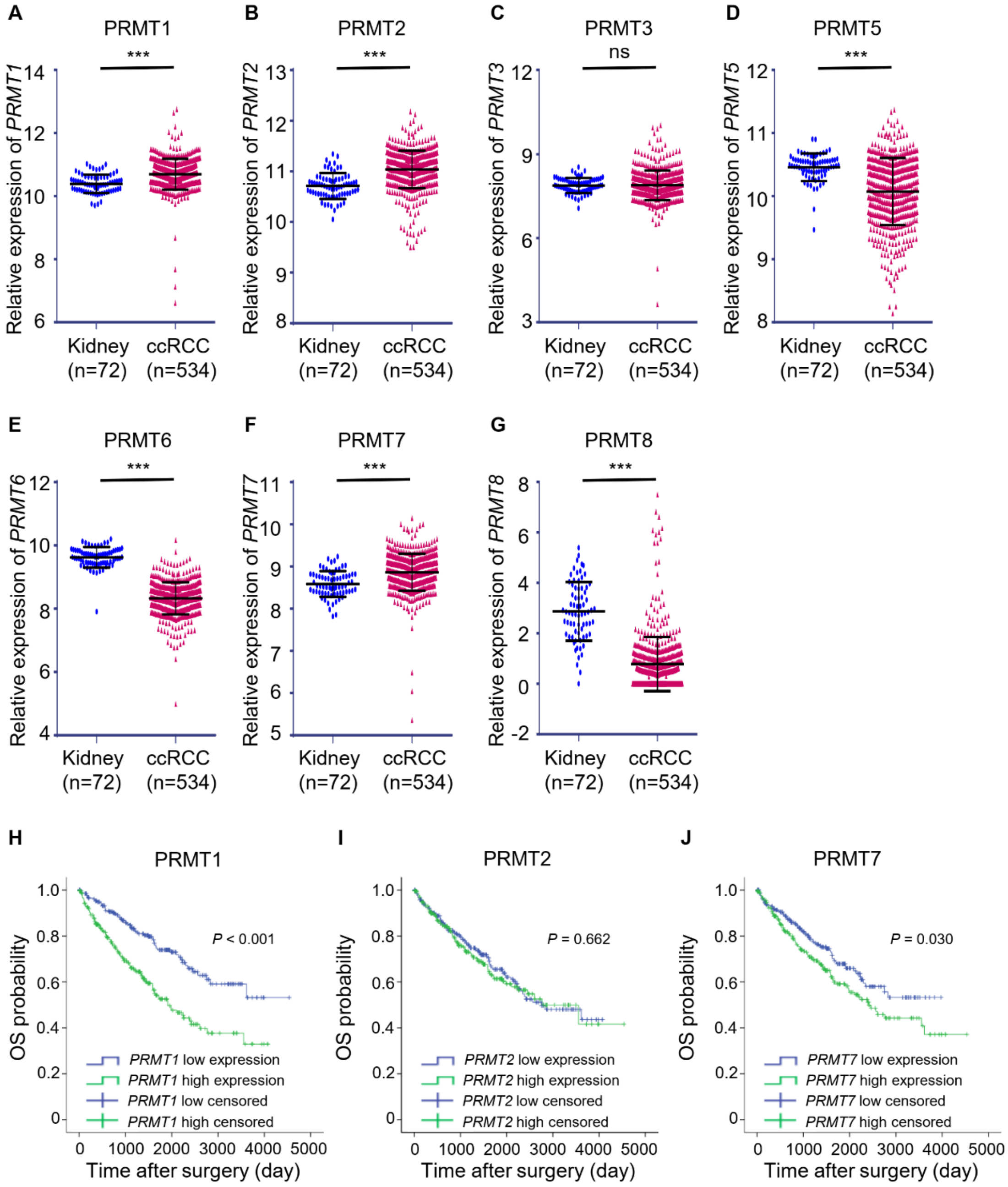

Figure S1. PRMT1 and PRMT7 expression levels were significantly correlated with the progression and prognosis of ccRCC patients. (A-G) The mRNA levels of *PRMTs* were analyzed in the ccRCC in TCGA dataset compared to the normal controls (*PRMT4* and *PRMT9* data were not included). **(H-J)** Kaplan–Meier analysis of overall survival for ccRCC patients with a low or high level of *PRMT1*, *PRMT2*, and *PRMT7* expression in TCGA dataset. ***$P < 0.001$.

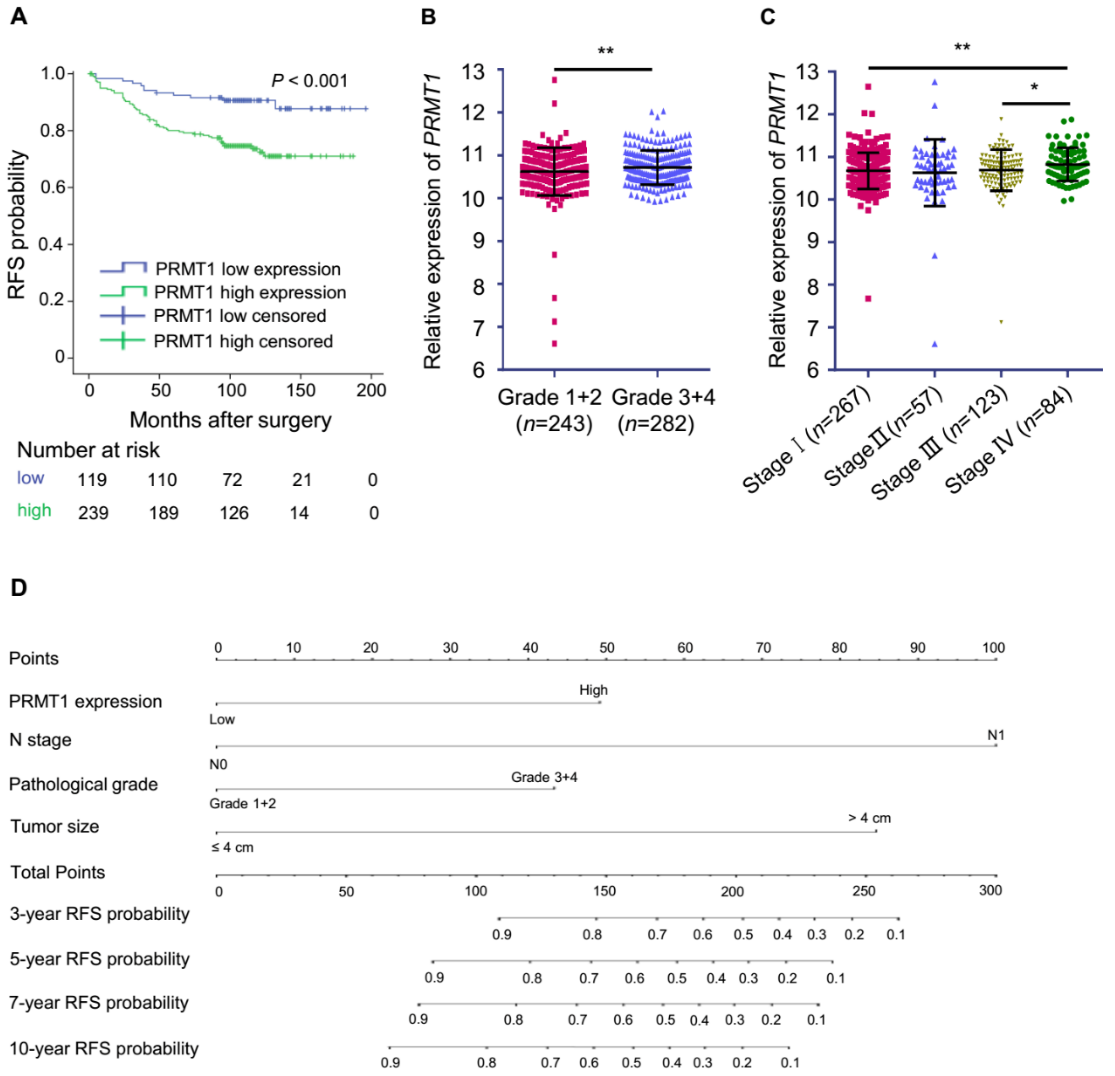

Figure S2. PRMT1 expression level is associated with the progression of ccRCC. (A) Kaplan–Meier curve of comparing RFS in different levels of PRMT1 expression groups (n = 119 in the low-expression group; n = 239 in the high-expression group) in Renji ccRCC TMAs. **(B-C)** The mRNA levels of PRMT1 expression in ccRCC were analyzed based on histological grade and clinical stage. **(D)** Nomogram for the prognosis of RFS in ccRCC patients from Renji ccRCC TMAs. *$P < 0.05$ and **$P < 0.01$.

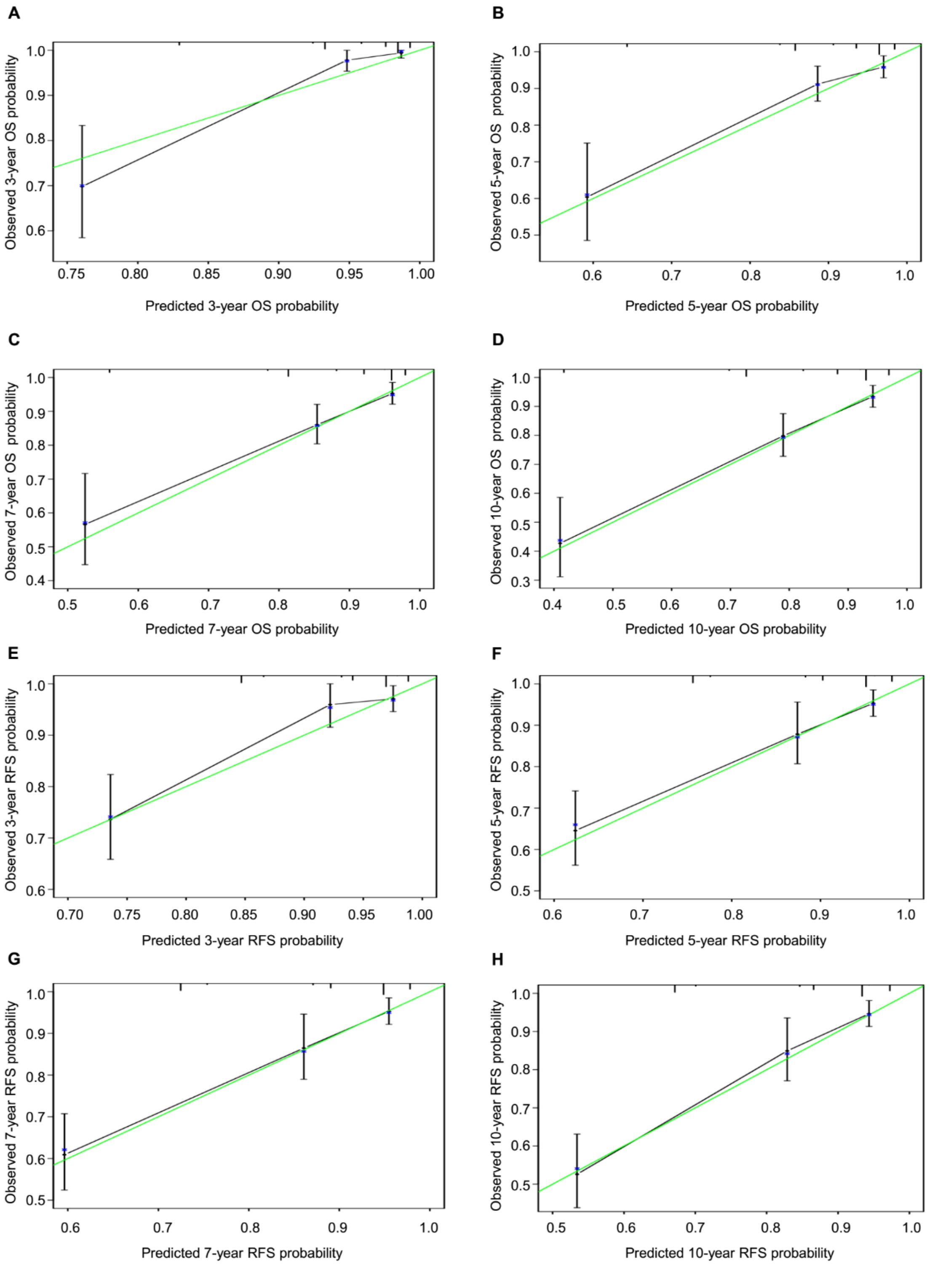

Figure S3. Calibration plots for the prognosis of OS and RFS in ccRCC patients from Renji ccRCC TMAs. (A) The calibration plot for OS at 3 years. **(B)** The calibration plot for OS at 5 years. **(C)** The calibration plot for OS at 7 years. **(D)** The calibration plot for OS at 10 years. **(E)** The calibration plot for RFS at 3 years. **(F)** The calibration plot for RFS at 5 years. **(G)** The calibration plot for RFS at 7 years. **(H)** The calibration plot for RFS at 10 years. The green line represents predicted survival probability; the black line represents actual survival probability.

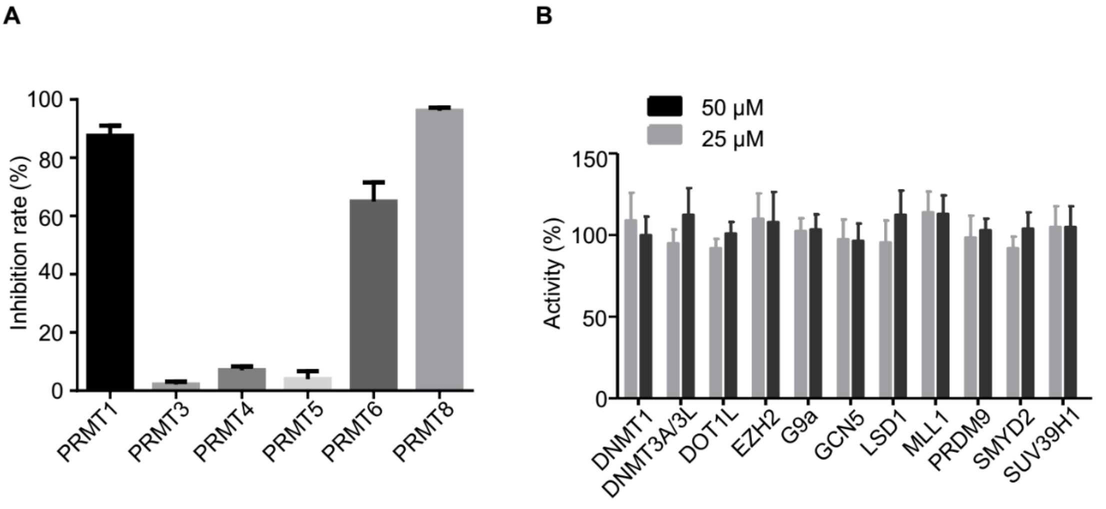

Figure S4. Selectivity of compound DCPT1061 against several epigenetic targets. (A) Inhibition rate of DCPT1061 (5 nM) against PRMT1, PRMT3, PRMT4, PRMT6, PRMT8, and PRMT5 detected based on using AlphaLISA method. **(B)** Inhibition of compound DCPT1061 against GCN5, DOT1L, EZH2, DNMT3A/3L, DNMT1, G9a, MLL1, PRDM9, SMYD2 and SUV39H1 at 25 μM and 50 μM.

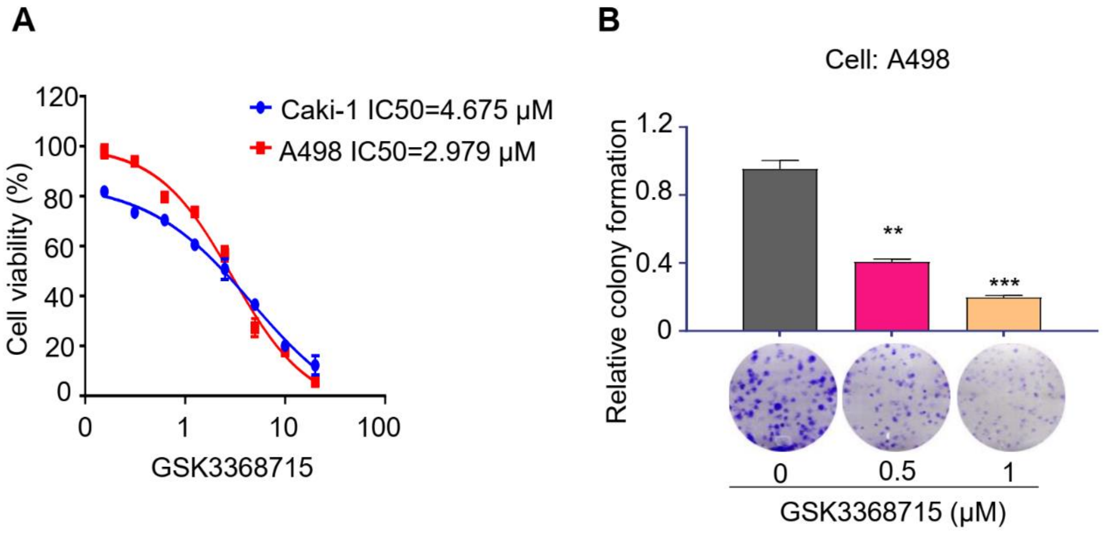

Figure S5. Type I PRMTs inhibitor GSK3368715 significantly inhibited ccRCC cell proliferation. **(A)** SRB assay was performed to determine the proliferation ability of ccRCC cells (786-O and A498) treated with GSK3368715 for 7 days in a dose-dependent manner. **(B)** Colony formation of A498 treated with different concentrations of GSK3368715.

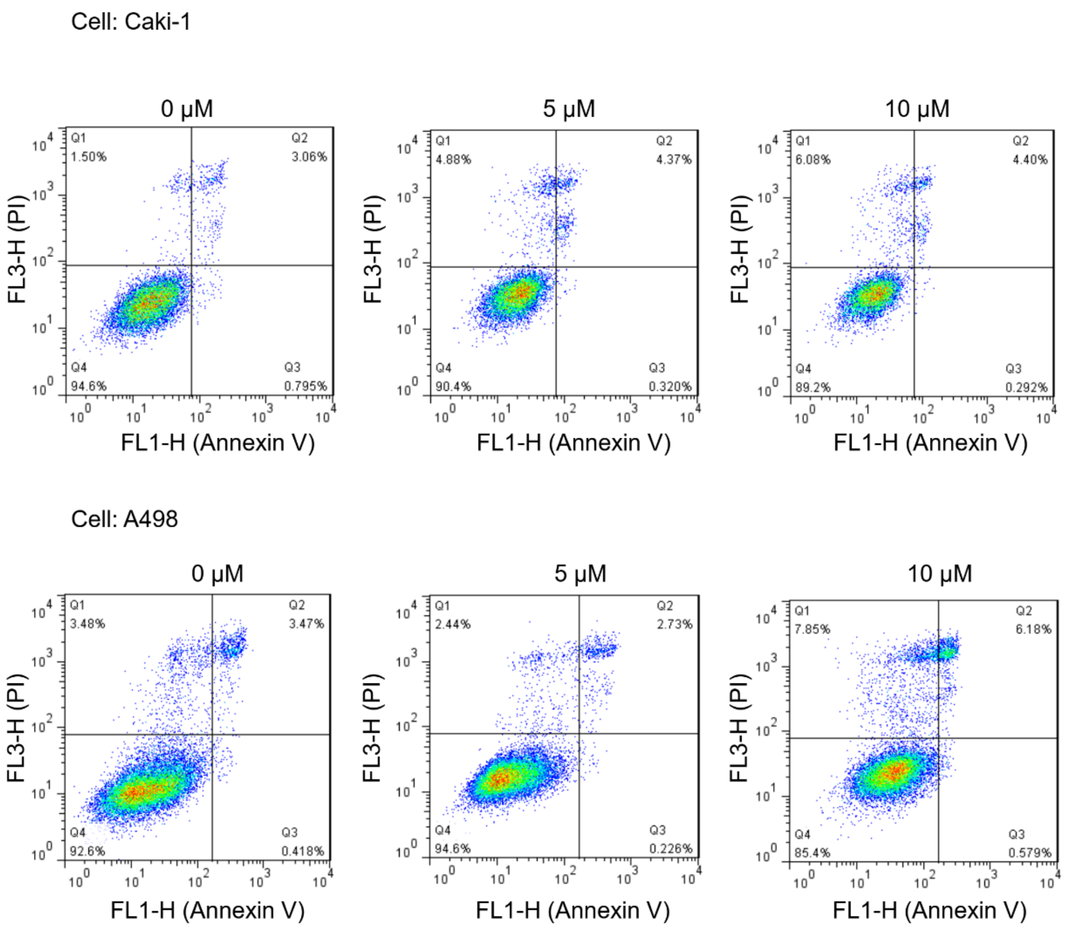

Figure S6. Treatment with DCPT1061 for 48 h barely impacts the apoptosis of ccRCC cells.

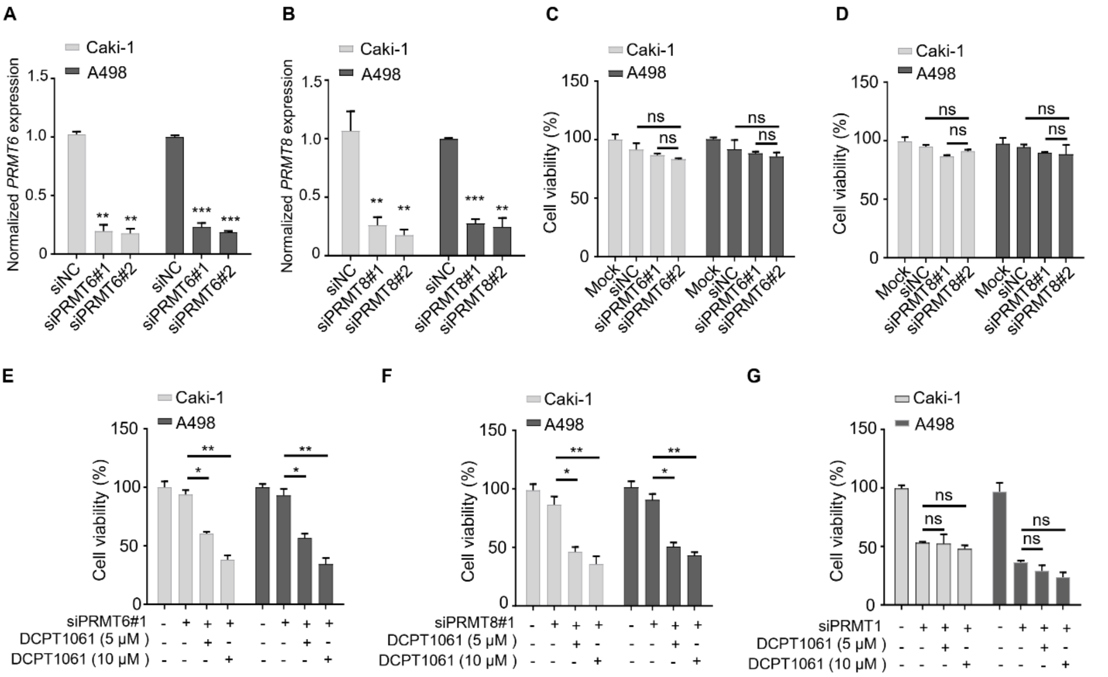

Figure S7. DCPT1061 suppresses the proliferation of ccRCC cells through the specific inhibition of PRMT1 instead of PRMT6 or PRMT8. (A-B) PRMT6 and PRMT8 mRNA levels were detected by RT-qPCR in siRNAs treated ccRCC cells (Caki-1 and A498) cells. **(C-D)** SRB assay was performed to evaluate the proliferation of PRMT6 or PRMT8-deleted ccRCC cells (Caki-1 and A498) cells. **(E-F)** SRB assay was performed to evaluate the proliferation of PRMT6 or PRMT8-deleted ccRCC cells (Caki-1 and A498) cells treated with DCPT1061. **(G)** SRB assay was performed to evaluate the proliferation of PRMT1-deleted ccRCC cells (Caki-1 and A498) treated with DCPT1061. **P* < 0.05, ***P* < 0.01, and ****P* < 0.001.

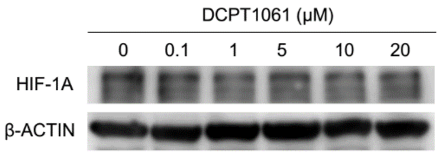

Figure S8. Correlation between PRMT1 inhibition and HIF-1A expression level in Caki-1 cells.

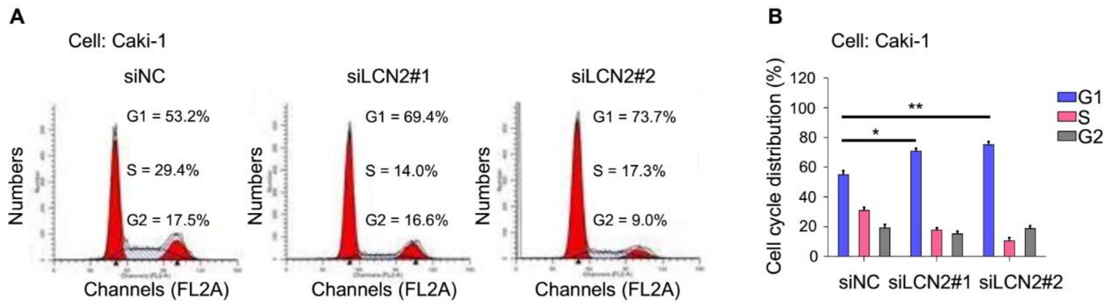

Figure S9. Knockdown of LCN2 significantly induced G1 cell cycle arrest in Caki-1 cells. *$P < 0.05$ and **$P < 0.01$.

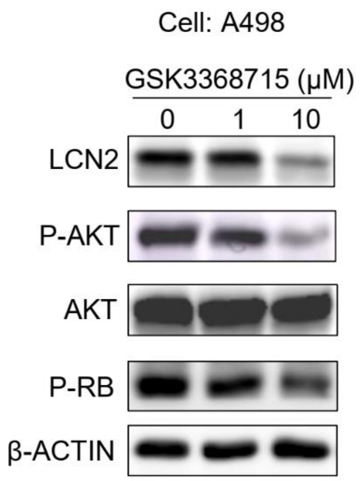

Figure S10. GSK3368715 treatment inhibits the LCN2-AKT-RB signal pathway in A498 cells.

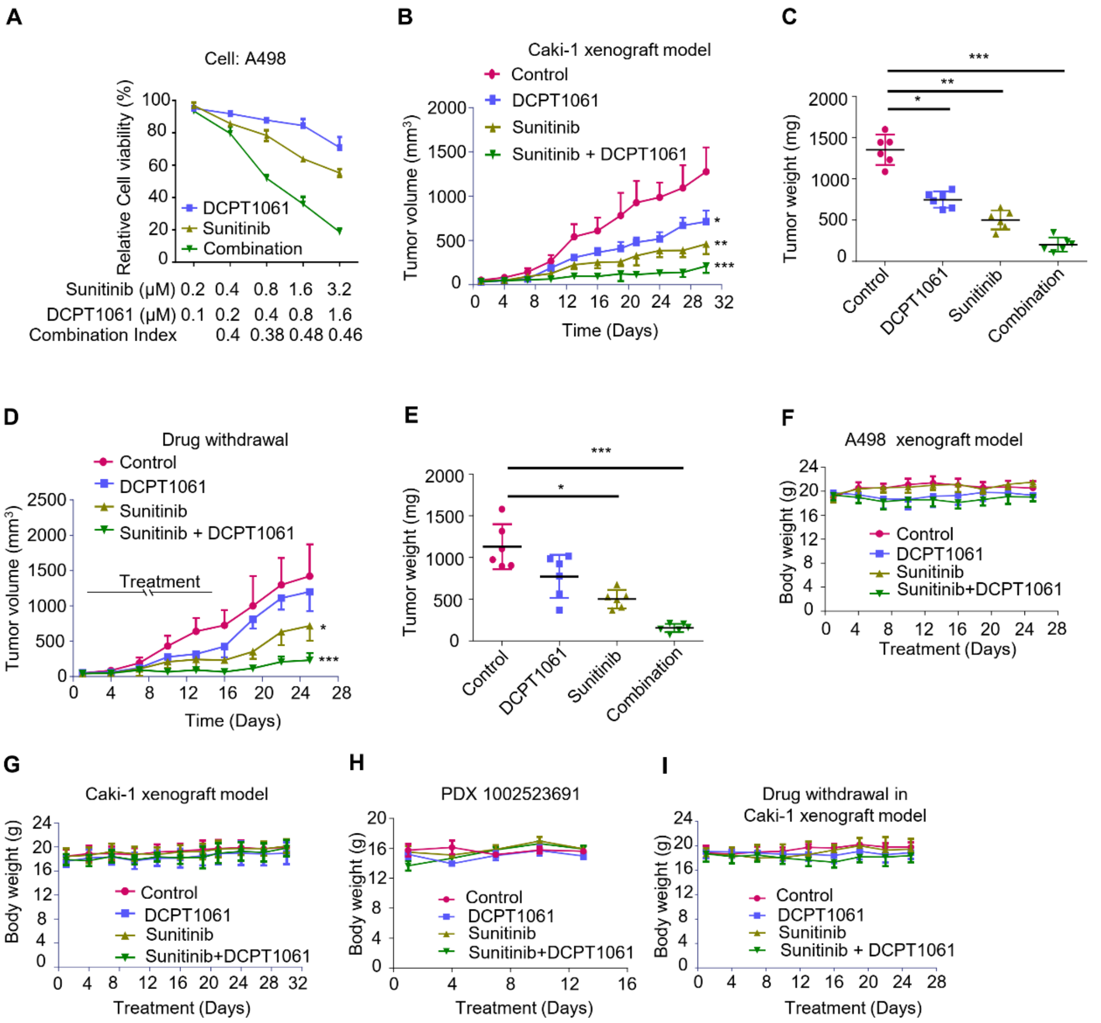

Figure S11. DCPT1061 and sunitinib combination treatment in ccRCC. (A) Cell proliferation data for A498 cells were accrued following 72 h exposure to the indicated compounds. Combination index (CI) was used to determine the synergistic effect of sunitinib and DCPT1061 treatment. **(B-C)** Tumor growth curves in the Caki-1 xenograft model, and tumor weights were expressed as mean ± SD, n = 6. **(D-E)** Drug withdrawal experiment after two weeks of treatment in Caki-1 xenograft models, and tumor weights were expressed as mean ± SD, n = 6. **(F-I)** Body weights of mice in A498, Caki-1, PDX 1002523691, and Caki-1 were expressed as mean ± SD, n = 6. *$P < 0.05$, **$P < 0.01$, and ***$P < 0.001$.

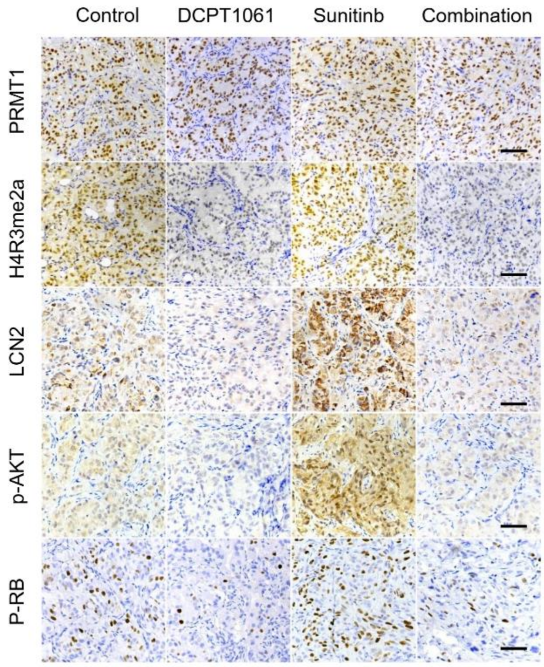

Figure S12. Representative staining of PRMT1, H4R3me2a, LCN2, p-AKT, and p-RB in the control, DCPT1061, sunitinib, and combination treated tumor tissues. (Bar: 100 µm).

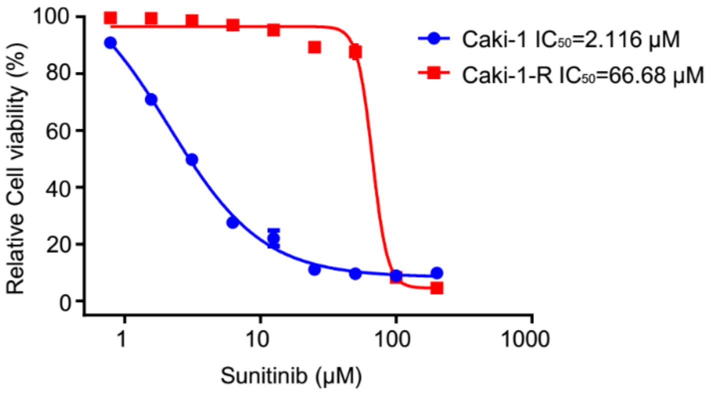

Figure S13. A sunitinib resistant ccRCC cell line (Caki-1-R) was constructed by exposing to sunitinib (30 μM, over 12 months), and SRB experiments were performed to detect the IC_{50} of sunitinib in Caki-1-R cells.

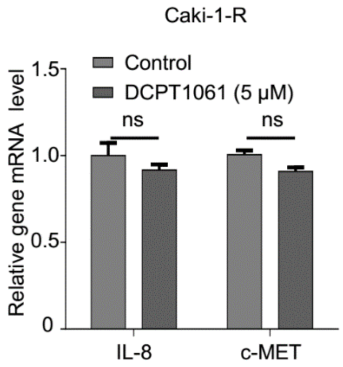

Figure S14. PRMT1 inhibition by DCPT1061 barely impacts the expression of *IL-8* and *c-MET* in Caki-1-R cells.
